# Growth differentiation factor 11 accelerates liver senescence through the inhibition of autophagy

**DOI:** 10.1111/acel.13532

**Published:** 2021-12-14

**Authors:** Jian Sun, Ying Li, Xiao Yang, Wei Dong, Jiankun Yang, Qi Hu, Cuntai Zhang, Haoshu Fang, Anding Liu

**Affiliations:** ^1^ Department of Biliopancreatic Surgery Sun Yat‐sen Memorial Hospital,Sun Yat‐sen University Guangzhou, Guangdong China; ^2^ Guangdong Provincial Key Laboratory of Malignant Tumor Epigenetics and Gene Regulation Sun Yat‐sen Memorial Hospital,Sun Yat‐sen University Guangzhou, Guangdong China; ^3^ Experimental Medicine Center Tongji Hospital, Tongji Medical College, Huazhong University of Science and Technology Wuhan, Hubei China; ^4^ Hepatic Surgery Center Tongji Hospital, Tongji Medical College, Huazhong University of Science and Technology Wuhan, Hubei China; ^5^ Hubei Key Laboratory of Hepato‐Pancreato‐Biliary Diseases Hubei Clinical Medicine Research Center of Hepatic Surgery Wuhan, Hubei China; ^6^ Key Laboratory of Organ Transplantation,Ministry of Education;NHC Key Laboratory of Organ Transplantation; Key Laboratory of Organ Transplantation Chinese Academy of Medical Sciences Wuhan, Hubei China; ^7^ Department of Geriatrics Tongji Hospital, Tongji Medical College, Huazhong University of Science and Technology Wuhan, Hubei China; ^8^ Department of Pathophysiology Anhui Medical University Hefei, Anhui China

**Keywords:** autophagy, hepatocyte, liver, senescence

## Abstract

The “rejuvenating” effect of growth differentiation factor 11 (GDF11) is called into question recently, and its role, as well as plausible signaling mechanisms in liver senescence, is unclear. To overexpress or knockdown GDF11, aged male mice are injected with a single dose of adeno‐associated viruses‐GDF11 or adenovirus‐small hairpin RNA‐GDF11, respectively. GDF11 overexpression significantly accelerates liver senescence in aged mice, whereas GDF11 knockdown has opposite effects. Concomitantly, autophagic flux is impaired in livers from GDF11 overexpression mice. Conversely, GDF11 knockdown increases autophagic flux. Moreover, rapamycin successfully restores the impaired autophagic flux and alleviates liver senescence in GDF11 overexpression mice, while the GDF11 knockdown‐mediated benefits are abolished by the autophagy inhibitor bafilomycin A1. GDF11 leads to a drop in lysosomal biogenesis resulting in defective autophagic flux at autophagosome clearance step. Mechanistically, GDF11 significantly activates mammalian target of rapamycin complex 1 (mTORC1) and subsequently represses transcription factor EB (TFEB), a master regulator of lysosomal biogenesis and autophagy. Inhibition of mTORC1 or TFEB overexpression rescues the GDF11‐impaired autophagic flux and cellular senescence. Hepatocyte‐specific deletion of GDF11 does not alter serum GDF11 levels and liver senescence. Collectively, suppression of autophagic activity via mTORC1/TFEB signaling may be a critical molecular mechanism by which GDF11 exacerbates liver senescence. Rather than a “rejuvenating” agent, GDF11 may have a detrimental effect on liver senescence.

## Graphical abstract

Model depicting the proposed mechanistic regulation of TFEB by GDF11. The role of GDF11 and its plausible signaling mechanisms is unveiled. GDF11 could increase the activity of mTORC1, resulting in reduced TFEB nuclear translocation and inhibition of lysosomal and autophagic genes transcription, thus accelerating liver senescence. These findings position GDF11 as an accelerator on liver senescence.

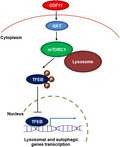

## 1 INTRODUCTION

Liver aging is usually associated with morphological and physiological changes, including a decline in hepatic blood flow, liver volume, and hepatic regenerative capacity (Schmucker, 2005). Aging may be related to an altered composition of the systemic milieu, and aging can be reversed or delayed by exposure to “young” systemic milieu via heterochronic parabiosis or injections of young plasma (Ashapkin et al., 2020). In line with this concept, the “rejuvenating” effect of young plasma is also observed in aged livers by us (Liu et al., 2018, 2019). Growth differentiation factor (GDF) 11, one of the factors identified in systemic milieu, has been shown to reduce age‐related cardiac hypertrophy of the heart (Loffredo et al., 2013; Poggioli et al., 2016), skeletal muscle and stem cell dysfunction (Sinha et al., 2014), and decline in neurogenesis and cognition (Katsimpardi et al., 2014). However, the “rejuvenating” effect of GDF11 has been questioned by several recent studies. GDF11 has no “rejuvenating” role in aged skeletal muscle satellite cells (Hinken et al., 2016) or on aging‐related pathological hypertrophy (Smith et al., 2015), but inhibits skeletal muscle regeneration (Egerman et al., 2015), decreases bone mass (Liu, Zhou, et al., 2016), induces the loss of cardiac and skeletal muscle mass and function (Hammers et al., 2017), and causes skeletal and cardiac muscle wasting (Zimmers et al., 2017). Consistently, a detrimental effect of GDF11 on liver injury was observed in our previous studies demonstrating that GDF11 worsened liver ischemia‐reperfusion injury and inhibited liver regeneration (Liu et al., 2018; Wang et al., 2019). Thus, further studies are needed to understand the contributions of GDF11 and its underlying mechanisms on liver senescence.

Autophagy is a homeostatic mechanism, degrades intracellular components via lysosome‐dependent machinery, thus plays critical role in the maintenance of protein quality control and adaptation to environmental challenges (Rubinsztein et al., 2012). Autophagy has a key role in cellular metabolism maintenance in the liver and is a beneficial mechanism against liver diseases (Czaja et al., 2013; Rautou et al., 2010). Aging causes a decline in autophagic activity which is linked to the damaged intracellular components accumulation and consequent cellular homeostasis disruption and dysfunction (Martinez‐Lopez et al., 2015; Rajawat et al., 2009; Rubinsztein et al., 2011). As demonstrated by us and others, autophagic activity is substantially impaired in the aged liver (Bi et al., 2020; Escobar et al., 2019; Liu et al., 2018, 2019). Restoration of the age‐impaired autophagic activity is able to improve cellular homeostasis and prevent or slow liver functional failure (Zhang & Cuervo, 2008). Transcription factor EB (TFEB), a transcriptional activator of lysosomal biogenesis and autophagic genes, is affected by its subcellular location. Phosphorylated TFEB locates in the cytoplasm under normal conditions. In response to starvation or other stressors, TFEB is dephosphorylated and transfer to the nucleus, it will activate its target genes associated with autophagosome formation, autophagosome‐lysosome fusion, cargo degradation, and lysosomal function (Sardiello et al., 2009; Settembre et al., 2011). TFEB nuclear localization, as well as its activity, is mainly mediated by serine phosphorylation which is regulated by the mechanistic target of rapamycin complex 1 (mTORC1) (Martina et al., 2012; Settembre et al., 2012). mTORC1 activation is dependent on its translocation from the cytosol to the lysosomal surface, which is mainly transduced via Rag GTPases. Rag GTPases, serving as a docking site for mTORC1, recruit mTORC1 to the lysosomal surface where mTORC1 is activated by Rheb GTPase (Bar‐Peled & Sabatini, 2014).

GDF11 may have a role on liver senescence, and insufficient autophagic activity is an important cause for liver senescence. This led us to hypothesize that GDF11 may modulate liver senescence via autophagy. To test this hypothesis, we investigated the pathophysiologic role of GDF11 on liver senescence, as well as its plausible signaling mechanisms in aged mice. We demonstrated that overexpression or knockdown of GDF11 accelerated or slowed liver senescence, which was linked to its ability to impair or improve autophagic activity, respectively. Mechanically, GDF11‐impaired TFEB‐mediated lysosomal biogenesis and autophagy via activating mTORC1, leading to a defective autophagic activity. Our findings reveal a detrimental effect of GDF11 on liver senescence and suppression of autophagic activity via mTORC1/TFEB may be a critical mechanism by which GDF11 accelerates liver senescence.

## 2 RESULTS

### 2.1 GDF11 accelerates liver senescence in aged mice

**FIGURE 1:**
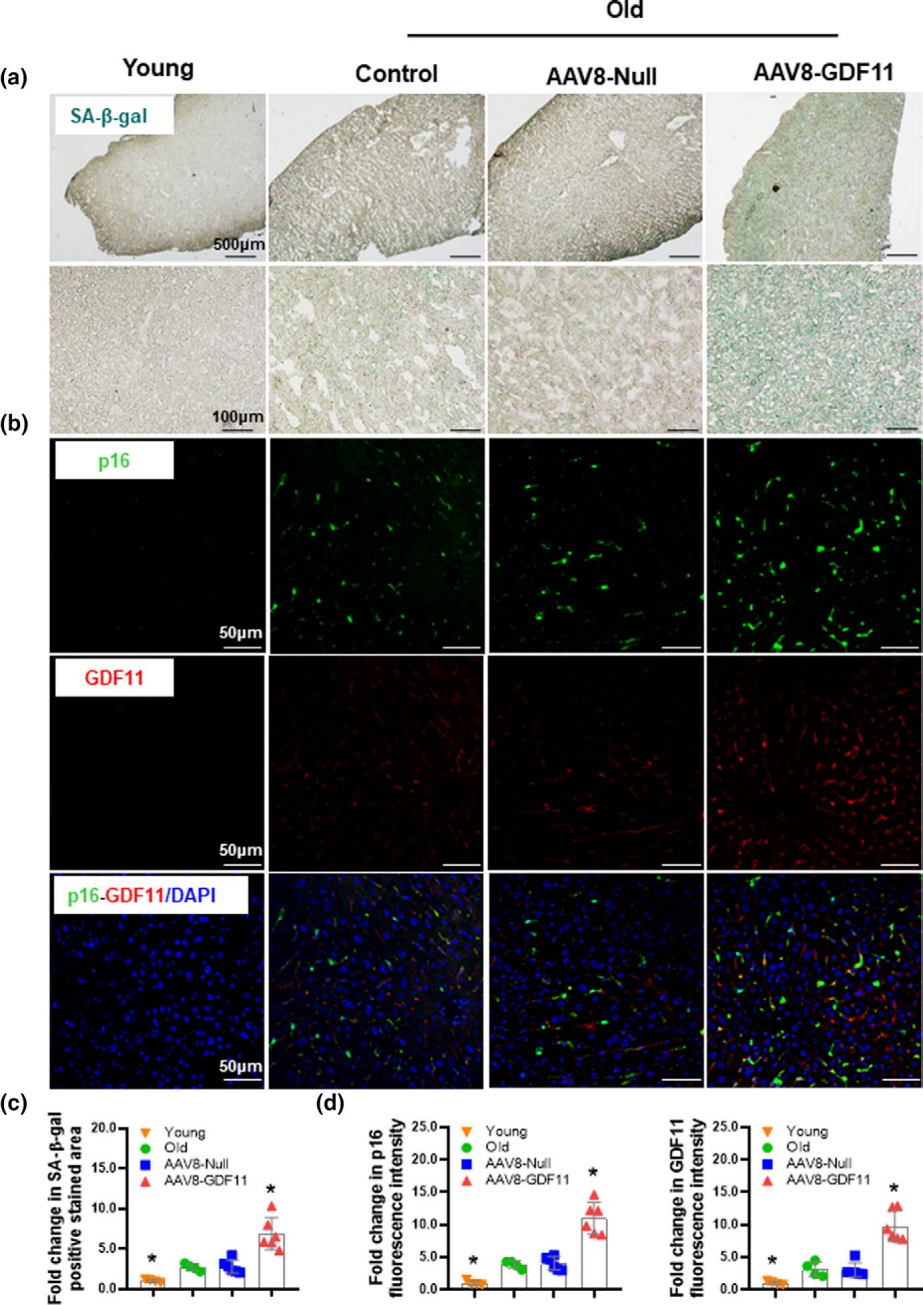
GDF11 exacerbates aging‐induced liver senescence. Aged male C57BL/6J mice (20 months) were injected intravenously with a single dose of AAV8‐GDF11 (5 × 10^11^ VG/mouse) for 8 weeks before detection. (a) Representative micrographs of SA‐β‐gal staining in hepatic tissues (original magnification, 40× or 400×). (b) Representative images of immunofluorescent staining for p16 or GDF11 in hepatic tissues (original magnification, 400×). (c) Percentage of SA‐β‐gal‐positive stained cell area normalized to young controls. (d) p16 or GDF11 fluorescence intensity normalized to young controls. The data are shown as mean ± *SD*, *n* = 4–6 per group, *p < 0.05 compared to old controls or the AAV8‐Null group

The GDF11's “rejuvenating” effect has been questioned and we previously demonstrated that GDF11‐inhibited liver regeneration (Liu et al., 2018, 2019). Thus, we evaluated whether GDF11 could affect liver senescence. Mice were injected with AAV8‐GDF11, and liver senescence was evaluated. Hepatic GDF11 mRNA expression levels were significantly increased after AAV8‐GDF11 injection. AAV8‐GDF11 also increased serum GDF11 protein levels (Figure S1). SA‐β‐gal activity, together with p16 INK4a (p16) expression, serves as the most widely used biomarker for detecting cellular senescence (Lee et al., 2006). As shown in Figure 1, AAV8‐GDF11‐treated mice displayed more SA‐β‐gal activity compared to that in the vehicle‐treated mice. p16 was almost undetectable in young livers. In aged livers, p16 expression was mainly detected in non‐parenchymal cells (NPCs). Weak staining for p16 was observed in hepatocytes. p16 staining was much stronger both in NPCs and hepatocytes in AAV8‐GDF11‐treated mice. Similarly, The GDF11 immunostaining pattern was consistent with p16. GDF11 also predominantly expressed in NPCs, and it is less expressed in hepatocytes in aged livers. AAV8‐GDF11 injection significantly increased GDF11 expression both in NPCs and hepatocytes. Furthermore, double immunofluorescent staining revealed the colocalization of p16 with GDF11. These findings indicate that GDF11 may accelerate liver senescence. Consistent with these observations, Western blot analyses showed that p16, as well as p21 Waf1/Cip1(p21) protein levels, were substantially upregulated in the liver from AAV8‐GDF11‐treated mice (Figure S2), whereas hepatic PCNA was significantly decreased in the AAV8‐GDF11‐treated mice, suggesting aggravation of liver senescence by GDF11 (Figure S2).

To further confirm the detrimental role of GDF11 in liver senescence, GDF11 was knocked down by Ad‐shRNA GDF11. Ad‐shRNA GDF11 injection substantially decreased hepatic GDF11 mRNA expression. Serum GDF11 levels were also significantly decreased in Ad‐shRNA GDF11‐injected mice (Figure S1). In contrast, GDF11 knockdown significantly delayed cellular senescence as indicated by less SA‐β‐gal accumulation and lower p16 and p21 protein levels, and improved liver proliferation as indicated by higher PCNA protein levels in the liver (Figure S2).

Then, the direct effect of GDF11 on hepatocytes was examined. Consistently, GDF11 upregulated SA‐β‐gal activity in primary cultured hepatocytes. GDF11‐treated hepatocytes showed higher expression level of p16 and p21, whereas lower expression of PCNA compared to that in the vehicle‐treated cells (Figure S3). Taken together, these observations suggest that GDF11 does not slow, but accelerates liver senescence.

### 2.2 GDF11 impairs autophagic flux

The contribution of autophagy to senescence has been well‐documented (Bi et al., 2020; Escobar et al., 2019). Indeed, Autophagy marker LC3B‐II and autophagic substrate p62 protein levels were significantly increased in livers obtained from mice treated with AAV8‐GDF11 compared to that in the vehicle‐treated mice. To better understand the impact of GDF11 on autophagic activity, LC3B and p62 levels were measured after treatment with chloroquine which potently blocks autophagosome‐lysosome fusion and impairs lysosomal acidification. Overexpression of GDF11 significantly inhibited liver autophagic flux, as indicated by substantial decreases in the chloroquine‐induced LC3B‐II and p62 accumulation (Figure S4c, d). In contrast, knockdown of GDF11 increased the accumulation of LC3B‐II and p62 by chloroquine, suggesting an increased liver autophagic flux (Figure S4e, f). Thus, GDF11 appears to be a negative regulator of the autophagic flux in the liver.

**FIGURE 2:**
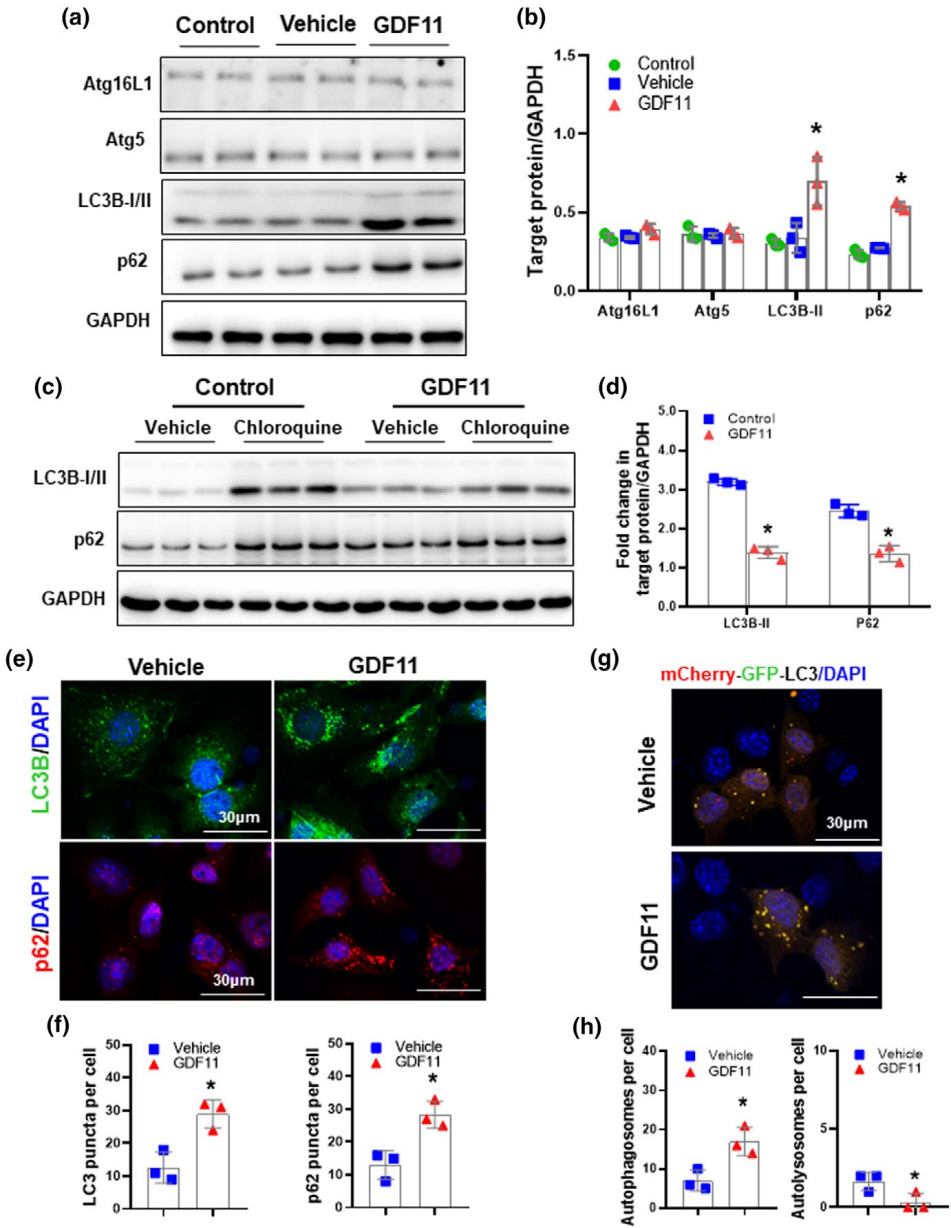
GDF11 impairs autophagic flux in hepatocytes. Primary hepatocytes were isolated from aged mice (20 months) and then cultured for 48 h in presence of GDF11 (100 ng/ml). (a) Western blot analysis of Atg16L1, Atg5, LC3B, and p62 protein expression. (b) Densitometric analysis of Atg16L1, Atg5, LC3B‐II, and p62. (c) Primary hepatocytes were treated with chloroquine (10 μM) for 6 h prior to detection. Western blot analysis of LC3B and p62 protein expression. (d) Densitometric analysis of LC3B‐II/GAPDH and p62/GAPDH ratio normalized to vehicle controls. (e) AML‐12 cells were cultured for 48 h in presence of GDF11 (100 ng/ml). Representative micrographs of LC3B and p62 staining in AML‐12 cells with or without GDF11 treatment (original magnification, 400×). (f) Quantification of the number of LC3B puncta and p62 puncta per cell. (g) After transient transfection with a mCherry‐GFP‐LC3 plasmid, AML‐12 cells were cultured for 48 h in presence of GDF11 (100 ng/ml). (h) Quantification of the number of LC3B puncta representing autophagosomes (yellow) and autolysosomes (red) per cell. The experiment was performed in triplicate with similar results. The data are shown as mean ± *SD*, *p < 0.05 compared to the vehicle group

We further observed that effect of GDF11 on autophagic activity in hepatocytes. As shown in Figure 2a, b, no significant changes were observed on Atg 5 and Atg16L1, whereas, LC3B‐II and p62 levels were significantly increased by GDF11. Chloroquine caused an accumulation of LC3B and P62 both in GDF11‐ and vehicle‐cultured hepatocytes. Moreover, the increases in LC3B‐II and P62 accumulation in GDF11‐cultured hepatocytes were significantly lower than that in the vehicle group (Figure 2c, d).

To corroborate these findings, we evaluated the variation in autophagosome formation using LC3B immunostaining. LC3B puncta formation was remarkably increased in GDF11‐cultured AML‐12 cells (Figure 2e, f). GDF11 also increased formation of p62‐positive autophagosomes (Figure 2e, f). Autophagy induction or/and its blockade may contribute to the increased number of autophagic vesicles (Menzies et al., 2012; Mizushima et al., 2010; Tanida et al., 2005). To directly evaluate autophagic flux via immunostaining, AML‐12 cells were transfected with a mCherry‐GFP‐LC3 tandem‐tagged reporter plasmid. GDF11 significantly inhibited autophagic flux in AML‐12 cells, as indicated by an increase in autophagosomes (yellow dots) and a decrease in autolysosomes (red dots) (Figure 2g, h). This finding further supports the concept that GDF11 inhibits autophagic activity in the liver.

### 2.3 GDF11 accelerates liver senescence via impairment of autophagy

**FIGURE 3:**
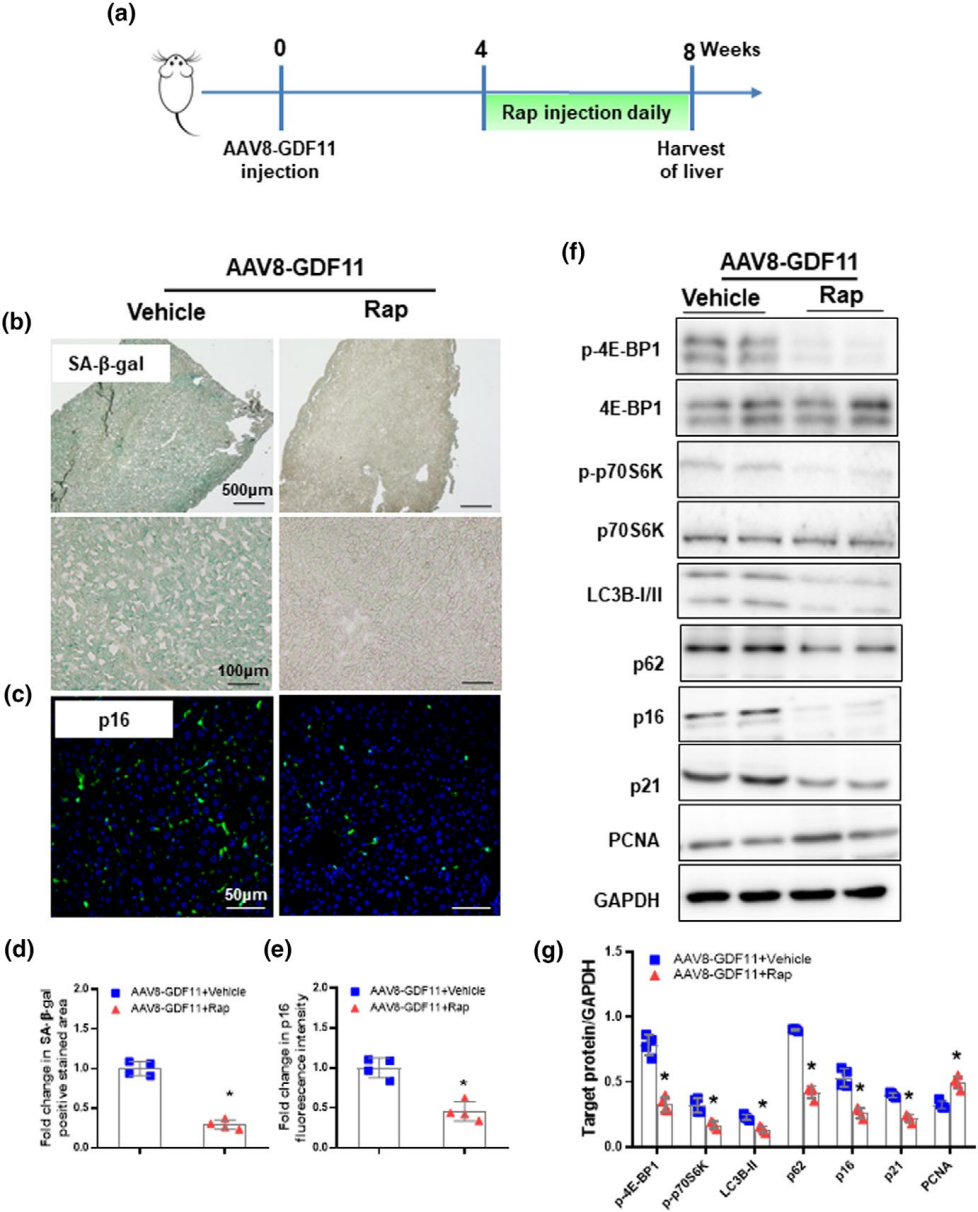
Overexpression of GDF11 accelerates liver senescence via impairment autophagic activity. (a) Experimental timeline: Aged male inbred C57BL/6J (20 months) mice were injected intravenously with a single dose of AAV8‐GDF11 (5 × 10^11^ VG/mouse) on week 0. Rapamycin (Rap, 1mg/kg, intraperitoneally) was given daily to the AAV8‐GDF11‐treated aged mice from week 4. On week 8, the mice were sacrificed and livers were harvested as indicated in the layout. (b) Representative micrographs of SA‐β‐gal staining in hepatic tissues (original magnification, 40× or 400×). (C) Representative images of immunofluorescent staining for p16 in hepatic tissues (original magnification, 400×). (d) Percentage of SA‐β‐gal‐positive stained cell area normalized to vehicle controls. (e) p16 fluorescence intensity normalized to vehicle controls. (f) Western blot analysis of p‐4E‐BP1, 4E‐BP1, p‐p70S6K, p70S6K, LC3B, p62, p16, p21, and PCNA protein expression. (g) Densitometric analysis of p‐4E‐BP1, p‐p70S6K, LC3B‐II, p62, p16, p21, and PCNA. The data are shown as mean ± *SD*, *n* = 4 per group, *p < 0.05 compared to the vehicle group

To investigate the contribution of autophagy to the GDF11‐induced liver senescence, we used rapamycin, an mTOR inhibitor that leads to autophagy activation, to restore impaired autophagic activity in the liver of AAV‐GDF11‐injected mice. Rapamycin‐treated mice showed reductions in hepatic LC3B‐II and p62 protein levels (Figure 3f, g). Rapamycin treatment abrogated the effect of GDF11 on the activity of SA‐β‐gal and p16 expression (Figure 3b–e). Furthermore, Western blot analyses showed that rapamycin inhibited the senescence marker p16, p21 protein levels (Figure 3f, g). Rapamycin rescued AAV8‐GDF11‐impaired liver proliferation as indicated by higher PCNA protein level (Figure 3f, g). Consistently, cellular senescence was also reversed by rapamycin in GDF11‐treated primary hepatocytes (Figure S5).

We used bafilomycin A1 to inhibit autophagy in the liver of Ad‐shRNA GDF11‐injected mice. Bafilomycin A1‐treated mice showed increases in hepatic LC3B‐II and p62 protein levels (Figure S6). Bafilomycin A1 abolished the effect of GDF11 knockdown in cellular senescence. Bafilomycin A1‐treated mice showed higher SA‐β‐gal activity, as well as p16 in livers than that in the vehicle control (Figure S6b–e). Consistently, bafilomycin A1 abolished the GDF11 knockdown‐induced decreases in p16 and p21, and increases in PCNA in livers (Figure S6f, g). Thus, GDF11 exacerbates the impairment of autophagic activity during senescence, which subsequently accelerates liver senescence.

### 2.4 GDF11 affects maturation/degradation of autolysosomes

**FIGURE 4:**
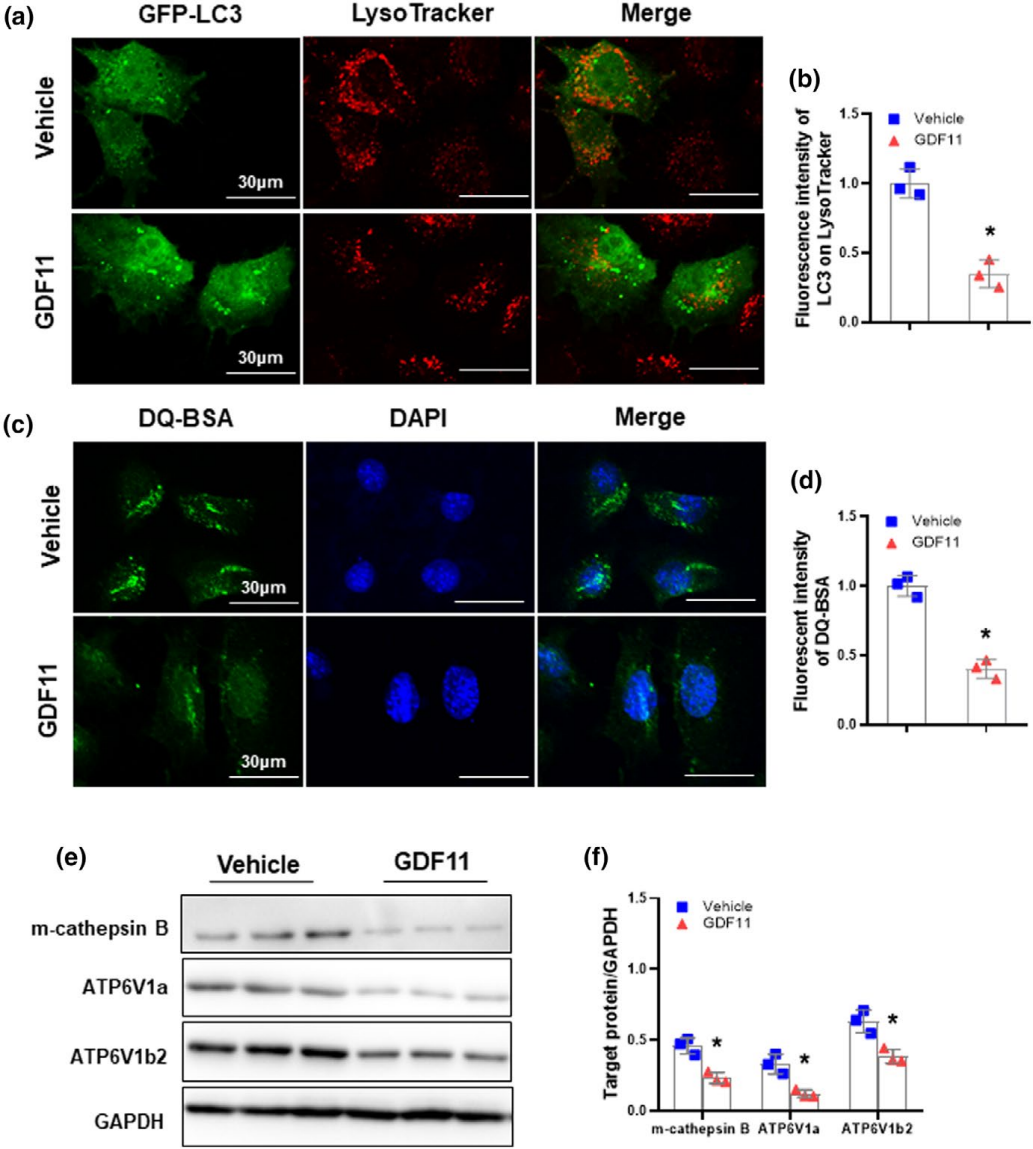
GDF11 decreases lysosomal function. (a) AML‐12 cells transient expressing GFP‐LC3 were cultured for 48 h in presence of GDF11 (100 ng/ml). After staining with LysoTracker Red DND‐99, cells were examined by confocal microscopy. Immunofluorescence confocal microscopy showing colocalization between LC3 puncta and LysoTracker Red with or without GDF11 (original magnification, 400×). (b) Quantification of the fluorescence intensity LC3 overlapping with Lysotracker signal normalized to vehicle controls. (c) Representative images of self‐quenched DQ‐BSA in AML‐12 cells (original magnification, 400×). (d) Quantification of the fluorescence intensity of DQ‐BSA normalized to vehicle controls. (e) Western blot analysis of m‐cathepsin B, ATP6V1a, and ATP6V1b2 protein expression. (f) Densitometry analysis of the m‐cathepsin B, ATP6V1a, and ATP6V1b2. The experiment was performed in triplicate with similar results. The data are shown as mean ± *SD*, *p < 0.05 compared to the vehicle group

It has been shown that blockade of the autophagic activity may result from the process of inhibition of autophagosome formation and/or impairment of lysosomal degradation (Menzies et al., 2012; Mizushima et al., 2010; Tanida et al., 2005). Because we observed that GDF11 had no effect in typical markers of autophagy initiation, including Atg5 and Atg16L1 (Figure 2a, b), we hypothesized that the defects in the later steps may contribute to the impairment of autophagic activity by GDF11. We transfected AML‐12 cells with a GFP‐LC3 plasmid, and colocalization of LC3 puncta (autophagosomal marker) with LysoTracker Red DND‐99 (lysosomal marker) was examined. We observed that lower colocalization of the two signals (yellow dots) in GDF11‐treated AML‐12 cells (Figure 4a, b). To further investigate the effect of GDF11 on lysosomes, we performed a bovine serum albumin (BSA) dequenching assay to monitor the degradative capacity of lysosomes (Vázquez & Colombo, 2009). Controls cells showed abundant signal of boron‐dipyrromethene fluorescent conjugate (BODIPY FL), whereas only weak signal was observed in GDF11‐cultured AML‐12 cells (Figure 4c, d). Western blots revealed that mature(m)‐cathepsin B, ATP6V1a, and ATP6V1b2 levels were decreased in GDF11‐cultured AML‐12 cells (Figure 4e, f), indicating that the proteolytic activity within lysosomes was decreased in response to GDF11. Consistently, AAV8‐GDF11‐inhibited lysosomal biogenesis as indicated by lower hepatic m‐cathepsin B, ATP6V1a, and ATP6V1b2 protein levels compared to that in controls (Figure S7). Conversely, GDF11 knockdown had opposite effects (Figure S8). These observations suggest that GDF11 may impair autophagic activity, likely due to a reduction in autophagosome‐lysosome fusion and/or an impairment in lysosomal degradation.

### 2.5 GDF11 inhibits lysosomal biogenesis in a TFEB‐dependent manner

**FIGURE 5:**
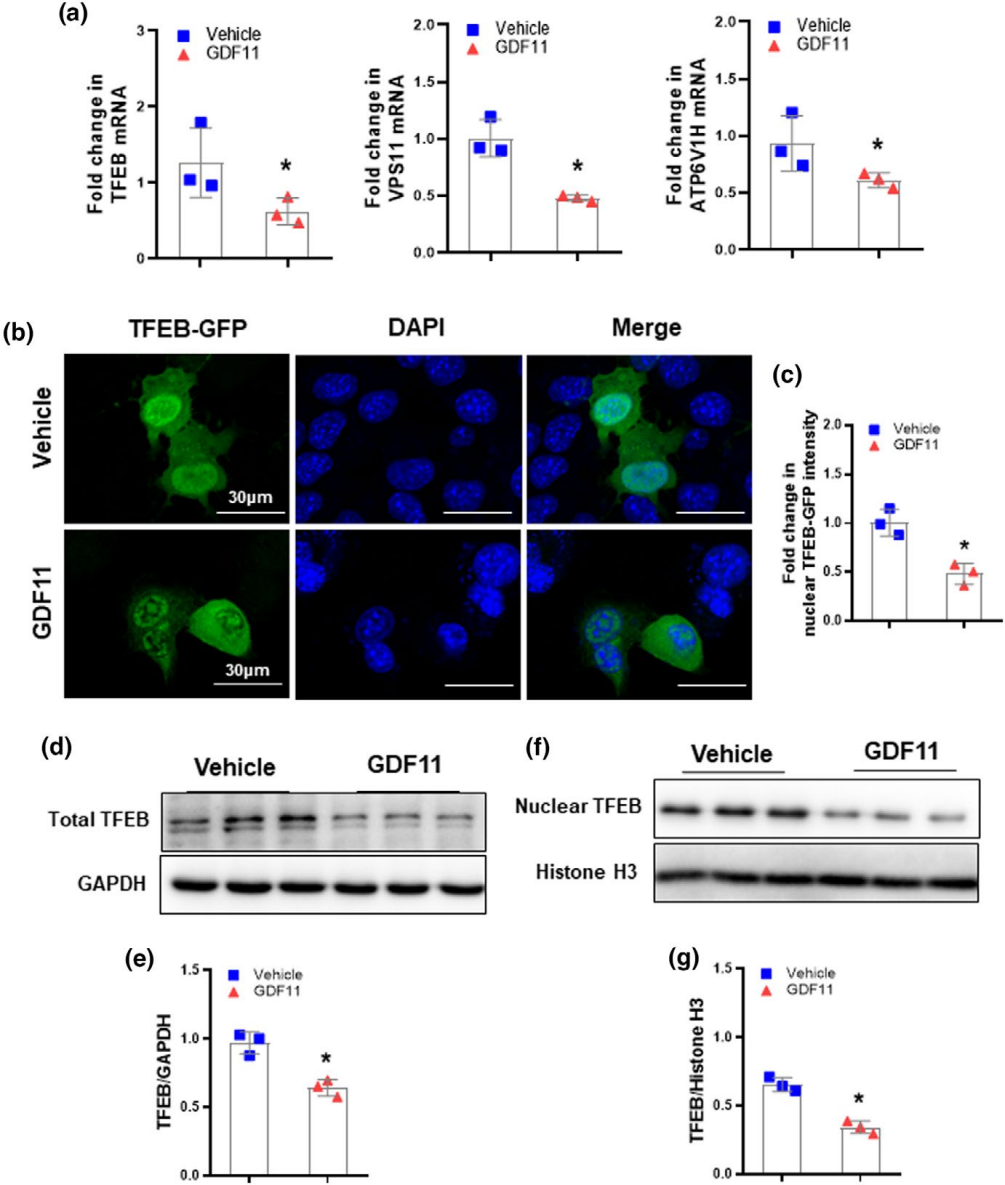
GDF11 inhibits TFEB activity. (a) AML‐12 cells were cultured for 48 h in presence of GDF11 (100 ng/ml). qRT‐PCR analysis of the mRNA levels of TFEB and its target genes VPS11 and ATP6V1H. (b) AML‐12 cells transient expressing GFP‐TFEB were cultured for 48 h in presence of GDF11 (100 ng/ml). Immunofluorescence confocal microscopy showing TFEB localization with or without GDF11 (original magnification, 400×). (c) Nuclear TFEB fluorescence intensity normalized to vehicle controls. (d) Western blot analysis of total TFEB protein expression. (e) Densitometry analysis of the total TFEB. (f) Western blot analysis of nuclear TFEB protein expression. (g) Densitometry analysis of the nuclear TFEB. The experiment was performed in triplicate with similar results. The data are shown as mean ± *SD*, *p < 0.05 compared to the vehicle group

As TFEB is a central regulator of lysosomal biogenesis and autophagy (Sardiello et al., 2009; Settembre et al., 2011), we speculated that GDF11 might reduce lysosomal biogenesis and autophagy through impairing TFEB activity. Due to TFEB promoter includes multiple CLEAR elements, TFEB can activate its own expression (Settembre et al., 2013). TFEB activity would generally be associated with its mRNA expression. Therefore, we evaluated TFEB mRNA levels as well as its target genes vacuolar protein sorting 11 (VPS11) and ATP6V1H both in GDF11‐treated hepatocytes and in vehicle‐treated control cells. We found that the mRNA expression of TFEB, VPS11, and ATP6V1H was lower in GDF11‐cultured hepatocytes than that in control cells (Figure 5a). TFEB activity can be also evaluated by its nuclear translocation (Settembre et al., 2011, 2012, 2013). AML‐12 cells were transiently transfected with a TFEB‐GFP construct. Immunofluorescence analysis showed GDF11 treatment reduced TFEB expression levels and inhibited TFEB nuclear translocation compared to that observed in control cells (Figure 5b, c). To confirm this finding, we evaluated total and nuclear TFEB by immunoblotting and revealed both lower total and unclear TFEB protein levels in GDF11‐cultured AML‐12 cells (Figure 5d–g). Furthermore, GDF11 overexpression decreased hepatic TFEB expression (Figure S7), whereas GDF11 knockdown increased TFEB expression (Figure S8).

To further confirm whether impaired activation of TFEB in response to GDF11 is a key mechanism contributing to the insufficient autophagy, we then transiently overexpressed TFEB to validate its regulatory role in GDF11‐impaired lysosomal biogenesis and autophagy. Exogenous expression of TFEB rescued TFEB activity in GDF11‐cultured AML‐12 cells (Figure S9a, b). Furthermore, TFEB overexpression restored the inhibitory effects of GDF11 on lysosomal biogenesis and autophagy, as evidenced by increases in lysosomal acidification and autophagic flux (Figure S9c–h). These data suggested that GDF11 impairs lysosomal biogenesis and autophagy which is likely due to its ability to inactivate TFEB.

### 2.6 GDF11 inhibits TFEB activity via mTORC1 signaling

It has been shown that phosphorylation is vital for TFEB subcellular localization, and it is known to be phosphorylated by mTORC1 or ERK2 (Martina et al., 2012; Roczniak‐Ferguson et al., 2012; Settembre et al., 2011, 2012). We therefore examined whether GDF11 could influence ERK2 and mTORC1 activity. We found that GDF11 had no effect on ERK1/2 activity by evaluating the phosphorylation of ERK1/2 levels compared with that of control cells (Figure S10). However, the phosphorylation of mTORC1 two well‐known downstream substrates, p70S6K1 and 4E‐BP1, were indeed elevated following GDF11 treatment (Figure S10). Furthermore, GDF11 treatment increased phosphorylation of AKT, reflecting AKT activation (Figure S10). Consistently, GDF11 overexpression significantly upregulated the phosphorylation level of AKT, p70S6K1, and 4E‐BP1 (Figure S11a, b). Conversely, knockdown of GDF11 had the opposite effects (Figure S11c, d). Thus, GDF11 appears to act as a positive mediator of mTORC1 activation in the liver.

**FIGURE 6:**
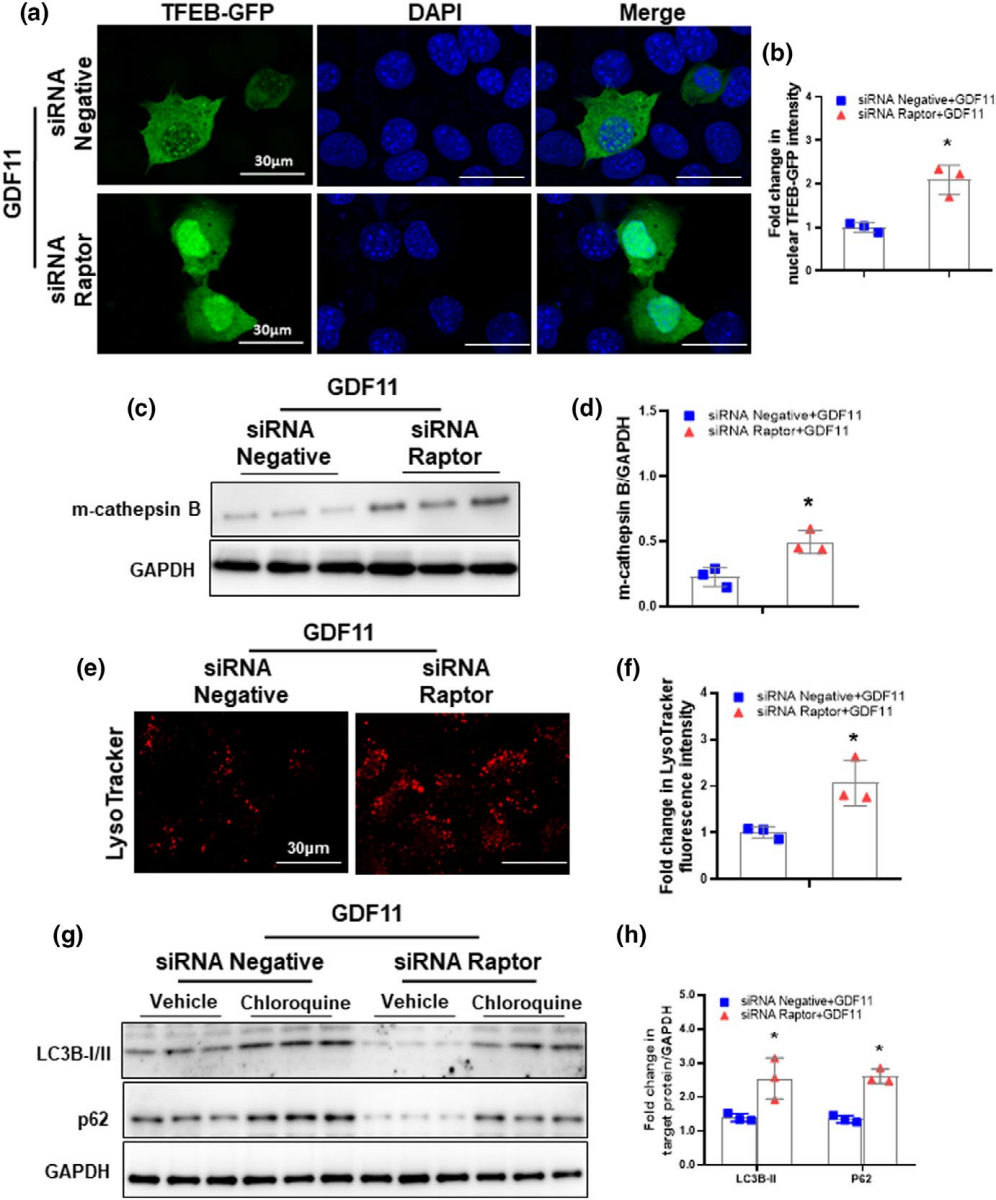
Raptor knockdown rescues lysosomal biogenesis and autophagy. (a) AML‐12 cells were transfected with siRNA‐negative or siRNA‐Raptor and then cultured for 48 h in presence of GDF11 (100 ng/ml). Immunofluorescence confocal microscopy showing TFEB localization in AML‐12 cells with or without siRNA‐Raptor (original magnification, 400×). (b) Nuclear TFEB fluorescence intensity normalized to controls. (c) Western blot analysis of m‐cathepsin B protein expression. (d) Densitometry analysis of m‐cathepsin B. (e) Representative images of LysoTracker staining in AML‐12 cells depleted of Raptor (original magnification, 400×). (f) LysoTracker signal intensity normalized to controls. (g) Western blot analysis of LC3B and p62 protein expression in AML‐12 cells depleted of Raptor with or without chloroquine (10 μM) for 6 h. (h) Densitometric analysis of LC3B‐II /GAPDH and p62/GAPDH ratio normalized to vehicle controls. The experiment was performed in triplicate with similar results. The data are shown as mean ± *SD*, *p < 0.05 compared to the vehicle group

To further determine whether GDF11 could inhibit TFEB activity via mTORC1, we genetically reduced mTORC1 activity by depleting Raptor (an essential component of mTORC1) with specific siRNA. Inhibition of GDF11‐induced mTORC1 activity by Raptor knockdown increased TFEB activity compared with that of control cells (Figure 6a, b). Furthermore, inhibition of mTORC1 activity could abrogate the inhibitory effect of GDF11 on lysosomal biogenesis and autophagy. The expression of m‐cathepsin B protein and LysoTracker staining of acidic vesicles was increased in Raptor siRNA‐transfected hepatocytes (Figure 6c–f). Furthermore, the accumulation of LC3B‐II and P62 by chloroquine in Raptor siRNA‐transfected hepatocytes was significantly higher than that in the vehicle group (Figure 6g, h). These data suggest that GDF11 can active mTORC1, thereby phosphorylates TFEB and subsequently inhibits autophagic activity.

### 2.7 Hepatocyte‐specific deletion of GDF11 does not affect lysosomal biogenesis, autophagy, and liver senescence

Because systemic germline GDF11 deletion (GDF11^−/−^) mice died within 24 h after birth, likely due to palate and renal anomalies (McPherron et al., 1999), we generated a mouse model in which GDF11 gene is specifically deleted in hepatocytes. GDF11 mRNA expression levels in livers obtained from GDF11^fl/fl^; Alb‐Cre mice were significantly decreased compared that in livers from GDF11^fl/fl^ controls (Figure S12a). To our surprise, serum GDF11 protein levels were almost identical between GDF11^fl/fl^; Alb‐Cre mice and GDF11^fl/fl^ controls (Figure S12b, c). Moreover, GDF11^fl/fl^; Alb‐Cre mice did not affect LC3B, p62, TFEB, and m‐cathepsin B levels (Figure S12d, e). These data suggest that hepatocyte‐specific deletion of GDF11 did not improve lysosomal biogenesis and autophagy. We further found that GDF11^fl/fl^; Alb‐Cre showed identical SA‐β‐gal accumulation, p16, p21, and PCNA protein level with GDF11^fl/fl^ controls (Figure S12d–g). Taken together, these results suggest specific deletion of hepatocyte GDF11 is not sufficient to improve lysosomal biogenesis and autophagy and slow liver senescence.

## 3 DISCUSSION

In addition to its essential roles in embryonic development, GDF 11 is also involved in other processes, such as aging. Recent findings have questioned GDF11 “rejuvenating” effect (Egerman et al., 2015; Hinken et al., 2016; Liu, Zhou, et al., 2016; Smith et al., 2015). The effect of GDF11 on liver senescence is unclear. The current study was designed to investigate the potential effects of GDF11 on liver senescence. The major and novel findings of this investigation are as follows: (a) GDF11 accelerates cellular senescence in hepatocytes and liver senescence in mice, while GDF11 knockdown is able to slow liver senescence; (b) the effect of GDF11 may be related to its ability to impair autophagic activity via modulating autophagosome processing; and (c) the impairment of autophagic flux may involve the activation of mTORC1 and subsequent the impairment of TFEB activity. These findings reveal previously undiscovered effect of GDF11 on liver senescence and provide new insights into its mechanisms related autophagic regulation.

To evaluate the pathophysiological role of GDF11 on liver senescence, we first overexpressed GDF11 in mouse livers by administration of AAV8‐GDF11. We demonstrated that AAV8‐GDF11 administration markedly increased GDF11 mRNA levels in the liver and protein levels in the serum. Moreover, GDF11 overexpression significantly accelerated liver senescence. Next, we knocked down GDF11 using an Ad‐shRNA approach. In contrast, GDF11 knockdown was able to slow liver senescence. The GDF11 immunostaining pattern was consistent with p16, and double immunofluorescent staining revealed the colocalization of p16 with GDF11, indicating GDF11 may accelerate liver senescence. We further generated hepatocyte‐specific deletion of GDF11 mice to determine the effect of GDF11on liver senescence. We demonstrated that GDF11 mRNA levels were significantly decreased in GDF11^fl/fl^; Alb^Cre^ mice. However, serum GDF11 protein levels in GDF11^fl/fl^; Alb^Cre^ mice were similar with that in their matched littermates. A similar observation has been reported previously by Garbern et al (Garbern et al., 2019). They showed that specific deletion of cardiomyocyte GDF11 was not sufficient to reduce GDF11 levels in the heart, as well as in the serum, respectively (Garbern et al., 2019). In this work, a ubiquitous promoter U6 was used for the Ad‐shRNA GDF11 construct which was injected intravenously via tail vein to knockdown GDF11 in the liver. It is possible that GDF11 in other organs, such as the lung, heart, skeletal muscle was also knocked down by Ad‐shRNA GDF11. Because approximately 80% of liver mass is composed of hepatocytes, and major sources of circulating GDF11 may not secrete from hepatocytes, as evidenced by the highest GDF11 mRNA levels observed in the spleen (Loffredo et al., 2013). Thereby, it is not unexpected that the amount of GDF11 in the serum are decreased in Ad‐shRNA GDF11‐injected mice, but not altered in GDF11^fl/fl^; Alb‐Cre mice. Furthermore, due to application of shRNA have unexpected off‐target effects that can impact several cellular phenotypes (Bofill‐De Ros & Gu, 2016; Rao et al., 2009), the Ad‐shRNA GDF11‐offered effects may also partially result from its off‐target effects. Hepatocyte‐specific deletion of GDF11 was not sufficient to slow liver senescence, suggesting compensatory effects of the other may buffer hepatocyte‐specific deletion of GDF11. Collectively, GDF11 may have a detrimental effect on senescence in the liver.

For complete autophagy maturation, autophagosomes need to fuse with lysosomes to form autolysosomes where the engulfed cargo is degraded (Glick et al., 2010; Klionsky & Emr, 2000; Rubinsztein et al., 2012). During the initiation of autophagy, the cytosolic form of LC3 (LC3‐I) is converted to LC3‐phosphatidylethanolamine conjugate (LC3‐II) once it conjugates to phosphatidylethanolamine. LC3‐II is then recruited to autophagosomal membranes and is degraded by lysosomal hydrolases when autophagosomes fuse with lysosomes. LC3‐II levels are associated with autophagosomes number prior to the formation of autolysosomes. In the case of GDF11, we showed that a significant increase in LC3B‐II protein. Increased LC3B‐II does not necessarily reflect high levels of autophagic activity, due to autophagosomes are intermediates in the autophagy process. Thus, the increase in LC3B‐II is either correlated with increased autophagosome formation or reduced autophagosome turnover (Menzies et al., 2012; Mizushima et al., 2010; Tanida et al., 2005). p62, a polyubiquitin binding protein, is selectively incorporated into autophagosomes through direct binding to LC3 and acts as one of specific substrates degraded by autophagosome‐lysosome fusion. Thus, its levels are inversely associated with autophagic activity and impaired autophagosome‐lysosome fusion results in p62 accumulation (Lippai & Low, 2014; Pankiv et al., 2007). Indeed, the accumulation in p62 was accompanied by LC3B‐II after GDF11 treatment, and the chloroquine‐induced LC3B‐II and p62 accumulation was inhbited by GDF11, indicating that autophagic flux is blocked by GDF11. Blockade of the autophagic flux may result from the process of inhibition of autophagosome formation and/or impairment of lysosomal degradation (Menzies et al., 2012; Mizushima et al., 2010; Tanida et al., 2005). Herein, GDF11 treatment significantly reduced autophagosome‐lysosome fusion, and also led to a drop in lysosome activation. These results suggest that GDF11 can inhibit lysosomal biogenesis, leading to insufficient autophagy.

These observations elicit an important question by which GDF11 impairs lysosomal biogenesis and consequently inhibits autophagy. Accumulation evidence indicates that TFEB is a master regulator for autophagy by driving genes expression which are involved in both early (autophagosome formation) and late (lysosome biogenesis) steps of autophagy (Sardiello et al., 2009; Settembre et al., 2011). Our data showed that GDF11‐inhibited TFEB activation was accompanied by impaired lysosomal biogenesis and autophagic flux, while TFEB overexpression rescued the GDF11‐induced impairment of lysosomal biogenesis and autophagy. TFEB activity is mainly regulated by several kinases, such as mTORC1, ERK2, and protein kinase Cβ (PKCβ), which can phosphorylate TFEB at specific amino acid residues. mTORC1 phosphorylates TFEB at serine 142, serine 11, and serine 122, resulting in TFEB cytosolic retention and subsequent proteasome‐mediated degradation (Martina et al., 2012; Roczniak‐Ferguson et al., 2012; Settembre et al., 2012). ERK2 phosphorylates TFEB at serine 142 and retains TFEB in the cytosol, and ERK2 inhibition induces TFEB nuclear translocation, whereas ERK2 induction reduces TFEB target gene expression (Settembre et al., 2011). In contrast, PKCβ phosphorylates TFEB at serine 461, serine 466, and serine 468, which stabilizes TFEB and increases its activity (Ferron et al., 2013). Mitogen‐activated protein kinase kinase kinase kinase (MAP4K3) phosphorylates TFEB at serine 3, which plays critical role in the interaction of TFEB with mTORC1‐Rag GTPase‐Ragulator complex and TFEB cytosolic sequestration (Hsu et al., 2018). We found that GDF11 can activate mTORC1 signaling. Genetic inhibition of Raptor caused the translocation of TFEB to the nucleus and reversed autophagic activity and lysosome function impaired by GDF11, further supporting that mTORC1 may be critical in GDF11‐induced inactivation of TFEB. In the current study, because GDF11 did not activate ERK1/2, ERK1/2 may be marginal in regulating GDF11‐induced inactivation of TFEB. Further research is needed to investigate whether and how GDF11 also affect MAP4K3 and PKC activity or others, which in turn regulates TFEB nuclear translocation and its activity. As one member of the TGF‐β superfamily, GDF11 binds to activin type IIA (ActRIIA) or type IIB (ActRIIB) and type I activin receptor‐like kinase (ALK) receptors ALK4, ALK5, and ALK7 to elicit signal transduction via SMAD2/3 (Andersson et al., 2006; Oh et al., 2002; Walker et al., 2016). A recent work has implicated that AKT1‐SMAD2/3 cross‐talk regulates cyclic mechanical stretch‐induced autophagy via primary cilia (Shim et al., 2021), and TGF‐β2/SMAD signaling is involved in the modulation of autophagy on epithelial‐mesenchymal transition in lens epithelial cells (Sun et al., 2021). Future studies will be conducted to investigate the effect of SMAD2/3 signaling in GDF11‐mediated autophagy. Collectively, these observations suggest that the GDF11‐regulated mTORC1/TFEB pathway may be involved in the regulation of autophagic activity.

In summary, we demonstrated that GDF11 could accelerate liver senescence, whereas the inhibition of GDF11 could slow liver senescence. The mechanism of action of GDF11 appears to relate its ability to activate mTORC1 signaling, which impairs hepatic TFEB‐mediated lysosomal biogenesis and consequently inhibits autophagic activity. Our observations suggest that rather than a “rejuvenating” agent, GDF11 may act as an accelerator on liver senescence during aging.

## 4 MATERIALS AND METHODS

### 4.1 Animal experiments

Animal care and experiments were performed according to procedures approved by the Animal Care and Use Committee of Huazhong University of Science and Technology. To selectively inactivate the GDF11 gene in liver, CRISPR‐Cas9 technology was used to insert loxP sites into the introns flanking exon 2 of the GDF11 gene in fertilized mouse eggs to generate GDF11 floxed (GDF11^fl^) mice (CKOCMP‐14561‐Gdf11‐B6J‐VA; Cyagen Biosciences). GDF11^fl/fl^ mice were crossed with Albumin Cre mice (Stock# 003574, The Jackson Laboratory) to generate liver‐specific GDF11 knockout (GDF11^fl/fl^; Alb‐Cre) mice. The GDF11^fl/fl^ littermates were used as controls. All mice were at the C57/BL6J genetic background. All mice were housed under specific pathogen‐free conditions with free access to water and food. The GDF11^fl/fl^; Alb‐Cre mice grew normally, their body weights are similar with littermate controls. Male inbred C57BL/6J mice were purchased from the Beijing HFK Bioscience. To determine the effect of GDF11 on liver senescence, aged male inbred C57BL/6J (20 months) mice were injected intravenously with a single dose of adeno‐associated virus serotype 8 (AAV8)‐GDF11 (5 × 10^11^ viral genomes (VG)/mouse, diluted to 100 μl in sterile phosphate‐buffered saline (PBS)), or adenovirus (Ad)‐small hairpin RNA (shRNA)‐GDF11 (1 × 10^9^ plaque‐forming unit (PFU)/mouse, diluted to 100 μl in sterile PBS) for 8 weeks before detection. AAV8‐GDF11, Ad‐shRNA‐GDF11, and control vectors were customized from Vigene Biosciences. Rapamycin (1 mg/kg, intraperitoneally, S1039; Selleck) or bafilomycin A1 (1 mg/kg, intraperitoneally, S1413; Selleck) was given daily to the AAV8‐GDF11‐ or Ad‐shRNA GDF11‐treated aged mice for 4 weeks before harvest, respectively. All of the animals received humane care according to the criteria outlined in the “Guide for the Care and Use of Laboratory Animals” prepared by the National Academy of Sciences and published by the National Institute of Health (NIH publication 86‐23, revised 1985).

### 4.2 Cell cultures and treatments

Primary mouse hepatocytes were isolated from old (20 months) male inbred C57BL/6J mice using a two‐step perfusion method as described previously (Liu, Huang, et al., 2016). The freshly obtained hepatocytes were suspended in Dulbecco's Modified Eagle Medium (DMEM, 11995065; Gibco) containing 10% of fetal bovine serum (FBS, 10100147; Gibco) and 100 IU/ml penicillin and 100 μg/ml streptomycin (10378016; Gibco). Following overnight incubation, cells were washed two times with PBS, and then incubated with various treatments in DMEM containing FBS and antibiotics. The differentiated non‐transformed mouse hepatocyte cell line alpha mouse liver 12 (AML‐12) (American Type Culture Collection) were cultured in DMEM/F12 medium (11320082; Gibco) supplemented with 10% FBS (Gibco), 1× insulin‐transferrin‐selenium‐G supplement (41400045; Gibco), 40 ng/ml dexamethasone (D4902; Sigma‐Aldrich), 100 IU/ml penicillin, and 100 μg/ml streptomycin (Gibco), and maintained at 37ºC in a humidified incubator containing 5% CO_2_ gas.

To determine whether GDF11 could affect hepatocyte senescence, primary hepatocyte isolated from old mice treated with GDF11 (100 ng/ml, 1958‐GD/CF; R&D Systems) for 48 h. To investigate whether the senescence effect of GDF11 was autophagy dependent, rapamycin (0.1 μM) was used to rescue the GDF11‐induced impairment of autophagic activity. In some experiments, mTORC1 activity was inhibited by depleting Raptor with specific small interfering RNA (siRNA) (Invitrogen), and TFEB activity was increased by overexpressing TFEB with Ad‐TFEB.

### 4.3 Immunohistochemistry

Immunohistochemistry was performed as previously described (Liu et al., 2018). Briefly, after deparaffinization in xylene and rehydration in graded ethanol, the sections were antigen retrieved in Tris/EDTA buffer (pH 9.0) for 20 min, incubated with 3% H_2_O_2_ for 5 min and blocked in 10% non‐immune serum for 30 min. The specimens were then incubated with a primary antibody against cathepsin B antibody (1:100, 12216–1‐AP; Proteintech) for 1 h. Negative controls were run in parallel by omitting the primary antibody. The intensity of cathepsin B staining was measured with Image J software (National Institutes of Health).

### 4.4 Immunofluorescence

After de‐waxing and rehydration, the liver sections were antigen retrieved in Tris/EDTA buffer (pH 9.0) for 20 min. After blocking in 10% non‐immune serum for 1 h, the sections were probed with a primary antibody against p16 (1:50, ab211542; Abcam) at 4°C overnight, washed three times with PBS and were incubated with an Alexa 488‐conjugated secondary antibody for 1 h at room temperature. For double immunofluorescence staining, the sections were then incubated with a primary antibody against GDF11 (1:200) at 4°C overnight and followed with an Alexa 647‐conjugated secondary antibody incubation. Nuclear staining was performed with 4’,6‐diamidino‐2‐phenylindole (DAPI).

Cells were rinsed with PBS and fixed in 4% paraformaldehyde for 15 min. The cells were then permeabilized with 0.2% Triton X‐100 in PBS for 5 min at room temperature. After nonspecific staining blocking, the cells were incubated with a primary antibody against microtubule‐associated protein 1 light chain (LC) 3B (1:200, ab48394; Abcam) or p62 (1:200, P0067; Sigma‐Aldrich) at 4°C overnight. After washing with PBS, the cells were subjected to incubation with Alexa Fluor secondary antibodies (1:500) for 1 h at room temperature. Nuclear staining was performed using DAPI. Negative controls were run in parallel by omitting the primary antibody. The specimens were photographed using an inverted Olympus FV1000 laser scanning confocal microscope (Olympus) at magnifications of 400×. For fluorescence intensity measurements, mean fluorescence was analyzed in randomly selected areas using the ImageJ software.

### 4.5 Transfection

GFP‐LC3, mCherry‐GFP‐LC3, and GFP‐TFEB plasmid were obtained from Dianjun Biosciences. AML‐12 cells were transiently transfected with plasmids using Lipofectamine 3000 reagent (L3000015; Invitrogen) following the manufacturer's protocol. The cells were cultured with indicated treatments after transfection for 24 h. After washing with PBS, the cells were fixed in 4% paraformaldehyde for 15 min, and nuclear staining was performed using DAPI. Immunofluorescent photographs were taken with an inverted Olympus FV1000 laser scanning confocal microscope (Olympus). AML‐12 cells were transfected with siRNA against mouse Raptor using Lipofectamine RNAiMAX reagent (13778‐075; Invitrogen) according to the manufacturer's instructions. A scrambled siRNA was used as negative control. The cells were treated with different conditions for further analysis after transfection for 48 h. For fluorescence intensity measurements, mean fluorescence was analyzed in randomly selected areas using the ImageJ software.

### 4.6 Senescence‐associated β‐galactosidase (SA‐β‐gal) staining

A commercially available senescence detection kit (CS0030; Sigma‐Aldrich) was used to evaluate senescence of hepatocytes according to the manufacturer's instructions. Briefly, cryosections or cells were fixed with fixative solution for 15 min at room temperature. After washing three times with PBS, the cryosections or cells were stained in β‐gal staining solution overnight at 37°C in a dry incubator (without CO_2_). The β‐gal‐stained area was measured with Image J software.

### 4.7 BSA dequenching assay

Cells were seeded on 4‐well chamber slides (154526; Thermo Fisher Scientific), cultured to 60%–80% confluency, and treated with GDF11 for 48 h. Cells were then incubated with Self‐quenched BODIPY FL conjugate of BSA (DQ‐BSA, 10µg/ml, 7932–2; BioVision) for 1 h. The cells were rinsed with three times in PBS and nuclear staining was performed using DAPI before microscopy analysis. Immunofluorescent photographs were taken with an inverted Olympus FV1000 laser scanning confocal microscope (Olympus). The fluorescence intensity was measured with Image J software.

### 4.8 Western blot analysis

Western blot analysis was performed as described previously (Liu et al., 2018; Wang et al., 2019). Total liver tissues or cultured cells lysates were prepared using radioimmunoprecipitation assay (RIPA) lysis buffer (AR0105; Boster) containing protease and phosphatase inhibitors (05892953001 and 05892970001; Roche). Nuclear and cytoplasmic fractions of cultured cells were isolated using a cytoplasmic/nuclear extraction kit following the manufacturer's protocol (78833; Thermo Fisher Scientific). Equal amounts of proteins were subjected to sodium dodecyl sulfate‐polyacrylamide gel electrophoresis (SDS‐PAGE) and then transferred to polyvinyldifluoride membranes (IPVH00010; Merck Millipore). After blocking, the membranes were probed with the following primary antibodies: anti‐GDF11 (1:1000, MAB19581; R&D Systems), anti‐p16 (1:1000), anti‐p21 (1:500; Proteintech, 10355‐1‐AP), anti‐proliferating cell nuclear antigen (PCNA, 1:1000, 2586; Cell Signaling Technology), anti‐autophagy‐related (Atg) 16L1 (1:1000, AP18176; Abgent), anti‐LC3B (1:1000), anti‐p62 (1:1000), anti‐Atg5 (1:500, 12994; Cell Signaling Technology), anti‐cathepsin B antibody (1:500), anti‐ATPase H+ transporting V1 subunit a (ATP6V1a, 1:1000, ab19270; Abcam), anti‐ATP6V1b2 (1:1000, ab73404; Abcam), anti‐TFEB (1:1000, H00007942‐M01; Novus Biologicals), anti‐extracellular signal‐regulated protein kinase 1/2 (ERK1/2, 1:1000, 9102; Cell Signaling Technology), anti‐phos‐ERK1/2 (Thr202/Tyr204) (1:1000, 9101; Cell Signaling Technology), anti‐70 kDa ribosomal protein S6 kinase 1(p70S6K1,1:2000, 2708; Cell Signaling Technology), anti‐phos‐p70S6K1 (T389) (1:1000, 9234; Cell Signaling Technology), anti‐eukaryotic initiation factor 4E‐binding protein 1 (4E‐BP1, 1:1000, 9452; Cell Signaling Technology), anti‐phos‐4E‐BP1 (Thr37/46) (1:1000, 2855; Cell Signaling Technology), anti‐AKT(1:1000, 9272; Cell Signaling Technology), anti‐phos‐AKT(Ser473) (1:1000, 4060; Cell Signaling Technology), anti‐Histone H3(1:1000, ab1791; Abcam), and anti‐GAPDH antibody (1:20,000; G9545, Sigma‐Aldrich). Western blots were acquired with the Kodak Image Station (Carestream Health Inc), and bands were quantified by densitometry using Image J software normalizing against GAPDH or Histone H3.

### 4.9 RNA isolation and quantitative real‐time polymerase chain reaction (qRT‐PCR)

Total RNA was extracted from snap‐frozen liver samples or cultured hepatocytes by TRIzol reagent (9109; Takara) and reverse‐transcribed into cDNA using the First‐Strand cDNA synthesis kit (RR036A; Takara). qRT‐PCR was performed in triplicate with a Roch Light cycle detection system (Roche) and a SYBR green master mix kit (RR820A; Takara) using specific primers. The specific primer sequences are as follows: GDF11 forward: 5'‐TGGCTGCTCCTAAGTTGTGG‐3’, reverse: 5'‐AGCACTCTAGGGCTTGAGGA‐3’; TFEB forward: CCAGAAGCGAGAGCTCACAGAT, reverse: TGTGATTGTCTTTCTTCTGCCG; ATP6V1H forward: ATGAGTACCGGTTTGCCTGG, reverse: GACTGAATGCCAGGAGCCAT; VPS11 forward: 5'‐TCCTTGCTCCCAGAGCCTAT‐3’; reverse: 5'‐GACAGGAAGACCACAGGCAA‐3’, LC3B forward: TGACCCAGCTTAAGCGACTG‐3’; reverse: AACCACATCCTAAGGCCAGC, p62 forward:5'‐GCTGAAGGAAGCTGCCCTAT‐3’; reverse:5'‐TTGGTCTGTAGGAGCCTGGT‐3’ and GAPDH forward: 5'‐TCAACAGCAACTCCCACTCTTCCA‐3’; reverse: 5’‐TTGTCATTGAGAGCAATGCCAGCC‐3'. GAPDH was served as internal references of genes. The 2^(−ΔΔCt)^ method was used for the relative quantification of target gene mRNA expression.

### 4.10 Statistical analysis

Statistical analysis was undertaken using SigmaStat software v3.5. Results are presented as the mean ± standard deviation (*SD*). One‐way analysis of variance (ANOVA) combined with Bonferroni's post hoc test was used to determine significant differences among groups. Differences were considered significant when *p*‐value below 0.05.

## CONFLICT OF INTEREST

None declared.

## AUTHOR CONTRIBUTIONS

Y.L., X.Y., J.S., W.D., and J.K.Y. performed research, collected, analyzed, and interpreted data. A.D.L. and J.S. conceived the study, designed research, and wrote the paper. Q.H., C.T.Z., H.S.F., J.S., and A.D.L. conceived the study, designed research, and revised the paper. All authors have read and approved the final manuscript.

## Supporting information

Fig S1‐S12

## Data Availability

The data that supports the findings of this study are available in the supplementary material of this article.
