# Supplementary material for: Growth differentiation factor 11 accelerates liver senescence through the inhibition of autophagy
Source: Aging Cell. 2021 Dec 14;21(1):e13532. doi: 10.1111/acel.13532 (PMC8761011; doi:10.1111/acel.13532)
Supplement: Supplementary file 1 — Fig S1‐S12 [file ACEL-21-e13532-s001.pdf]

Supporting information

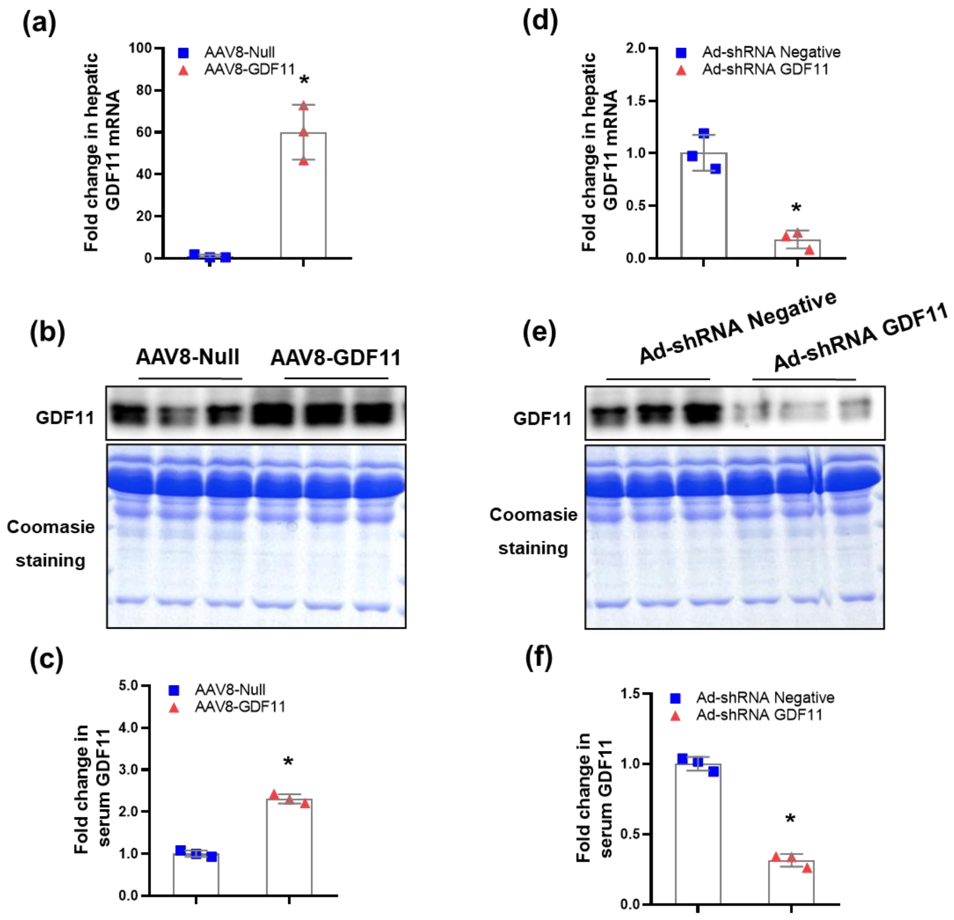

FIGURE S1. Analysis of GDF11 expression in AAV8-GDF11- or Ad-shRNA GDF11-injected mice. Aged male C57BL/6J mice (20 months) were injected intravenously with a single dose of AAV8-GDF11 (5 x 10^{11} VG/mouse) or Ad-shRNA GDF11 (1×10^{9} PFU/mouse mouse) for 8 weeks before detection. (a, d) qRT-PCR analysis of hepatic GDF11 mRNA expression normalized to AAV8-Null-injected mice or to Ad-shRNA Negative-injected mice. (b, e) Western blot analysis of serum GDF11 protein expression; Coomassie staining (bottom) demonstrates equivalent loading of each lane. (c, f) Densitometric analysis of serum GDF11 normalized to AAV8-Null-injected mice or to Ad-shRNA Negative-injected mice. The data are shown as mean ± SD. n=3 per group. *$p<0.05$ compared to the AAV8-GDF11 or Ad-shRNA Negative group.

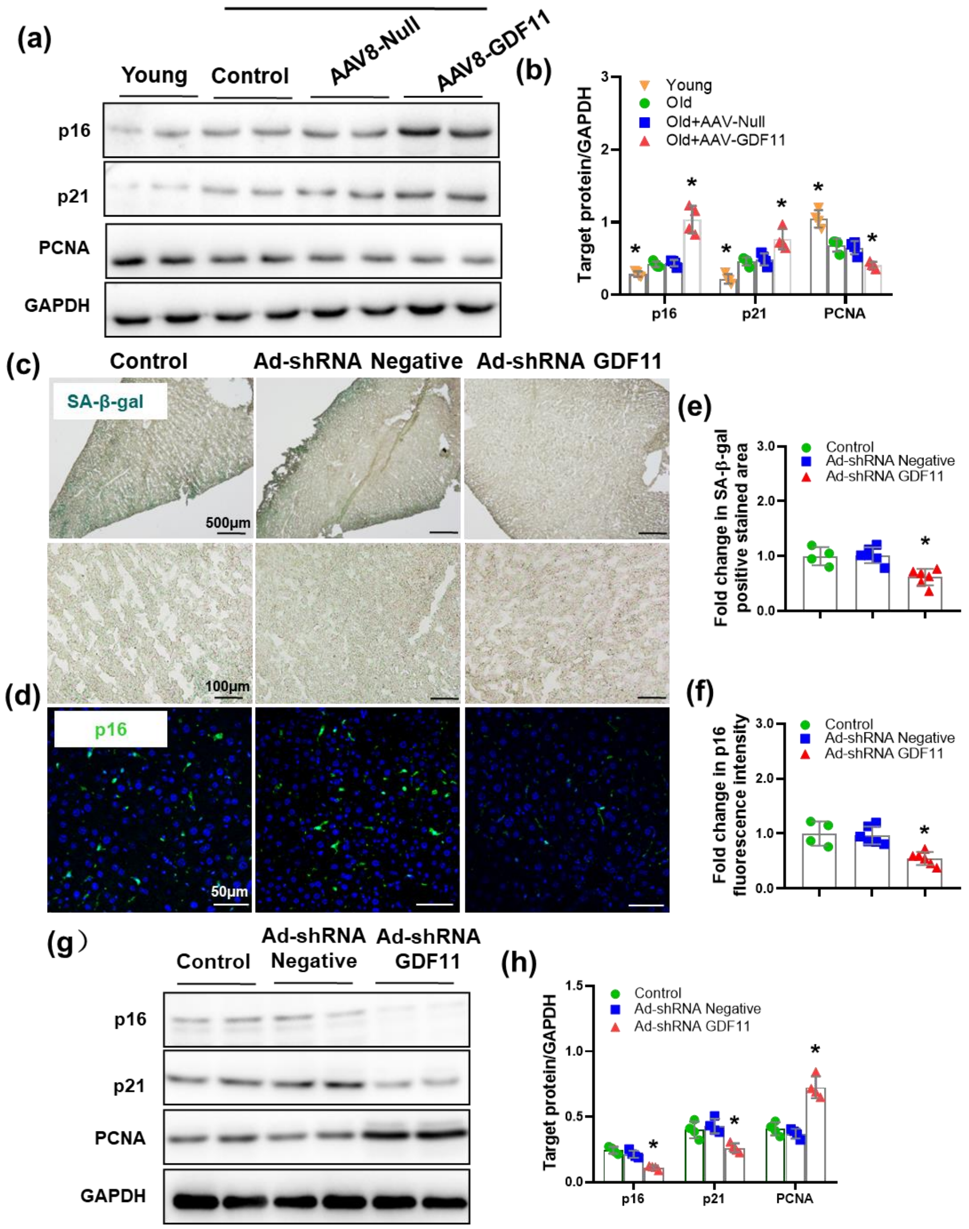

FIGURE S2. Overexpression or knockdown of GDF11 accelerates or slows aging-induced hepatic senescence. Aged male C57BL/6J mice (20 months) were injected intravenously with a single dose of AAV8-GDF11 (5 x 10^{11} VG/mouse) or Ad-shRNA GDF11 (1×10^{9} PFU/mouse mouse) and sacrificed after 8 weeks. (a) Western blot analysis of p16, p21 and PCNA protein expression in livers. (b) Densitometric analysis of p16, p21 and PCNA. (c) Representative micrographs of SA-β-gal staining in hepatic tissues (original magnification, 40 x or 400 x). (d) Representative images of immunofluorescent staining for p16 in hepatic tissues

(original magnification, 400 x). (e) Percentage of SA-β-gal-positive stained cell area normalized to controls. (f) p16 fluorescence intensity normalized to controls. (g) Western blot analysis of p16, p21 and PCNA protein expression in livers. (h) Densitometric analysis of p16, p21 and PCNA. The data are shown as mean ± SD, n = 4-6 per group, *p<0.05 compared to old controls, the AAV8-GDF11 or Ad-shRNA Negative group.

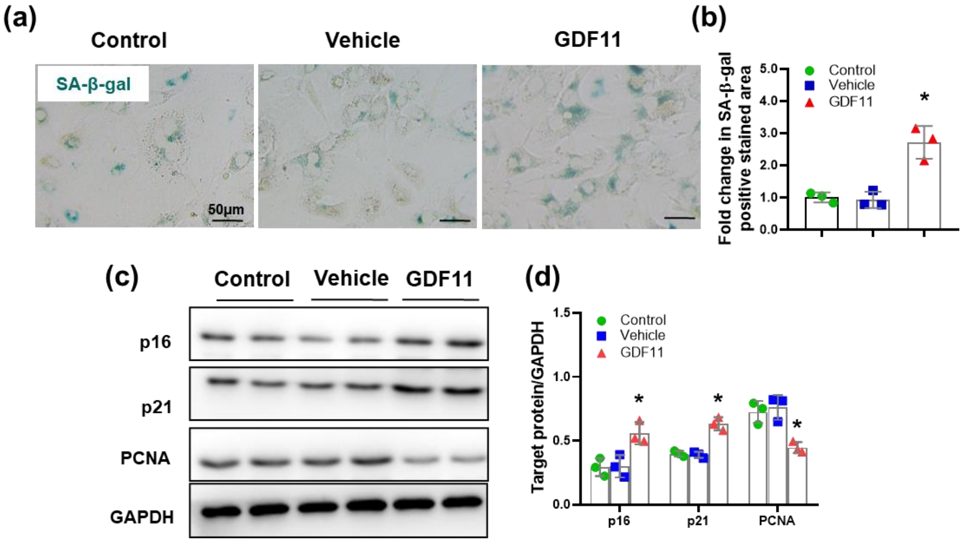

FIGURE S3. GDF11 induces cellular senescence. Primary hepatocytes were isolated from aged male C57BL/6J mice (20 months) and then cultured for 48 hours in presence of GDF11 (100 ng/ml). (a) Representative micrographs of SA-β-gal staining in hepatocytes (original magnification, 400 x). (b) Percentage of SA-β-gal-positive stained cell area normalized to controls. (c) Western blot analysis of p16, p21 and PCNA protein expression. (d) Densitometric analysis of p16, p21 and PCNA. The experiment was performed in triplicate with similar results. The data are shown as mean ± SD, *p<0.05 compared to the vehicle group.

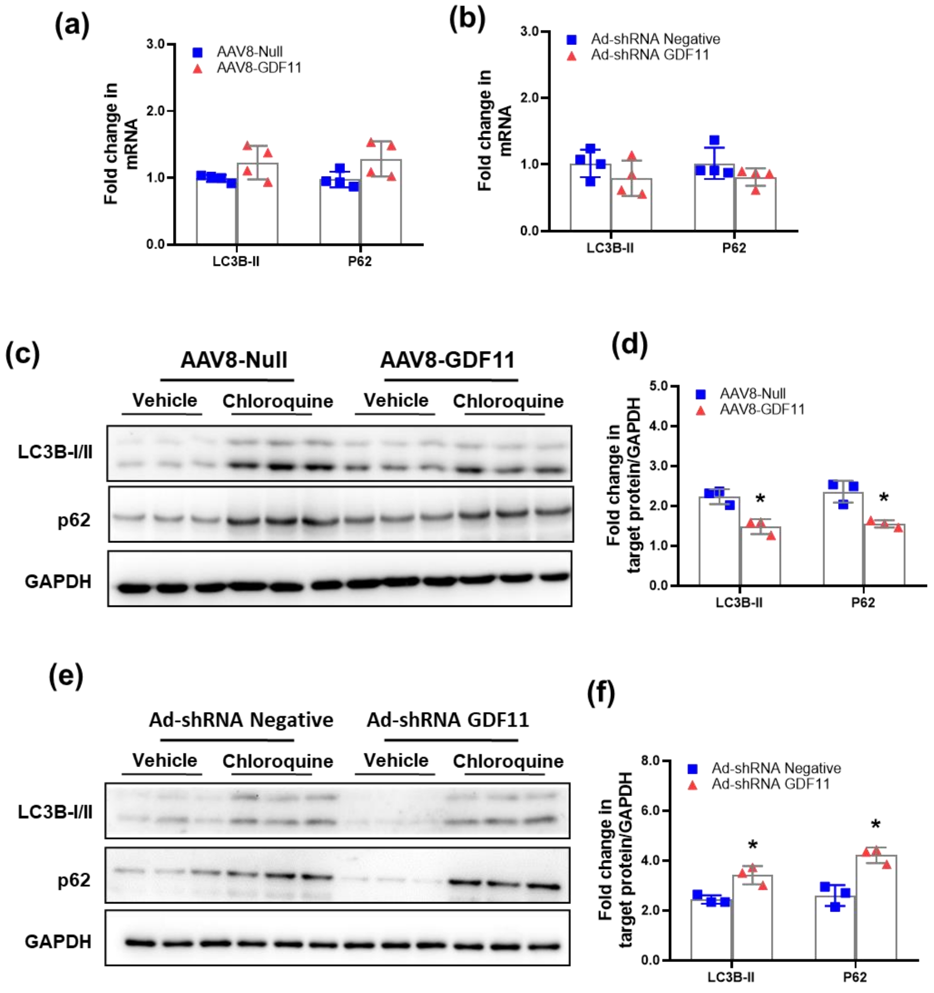

FIGURE S4. Analysis of autophagic flux in the liver from AAV8-GDF11- or Ad-shRNA GDF11-injected mice. Aged male C57BL/6J mice (20 months) were injected intravenously with a single dose of AAV8-GDF11 (5 x 10^{11} VG/mouse) or Ad-shRNA GDF11 (1×10^9 PFU/mouse mouse) and sacrificed after 8 weeks. (a, b) qRT-PCR analysis of hepatic LC3B and p62 mRNA expression normalized to AAV8-Null-injected mice or to Ad-shRNA Negative-injected mice. (c, e) Western blot analysis of LC3B and p62 protein expression with or without chloroquine (60 mg/kg). (d, f) Densitometric analysis of LC3B-II/GAPDH and p62/GAPDH ratio normalized to vehicle controls. The data are shown as mean ± SD. n=3-4 per group. *p<0.05 compared to the vehicle group.

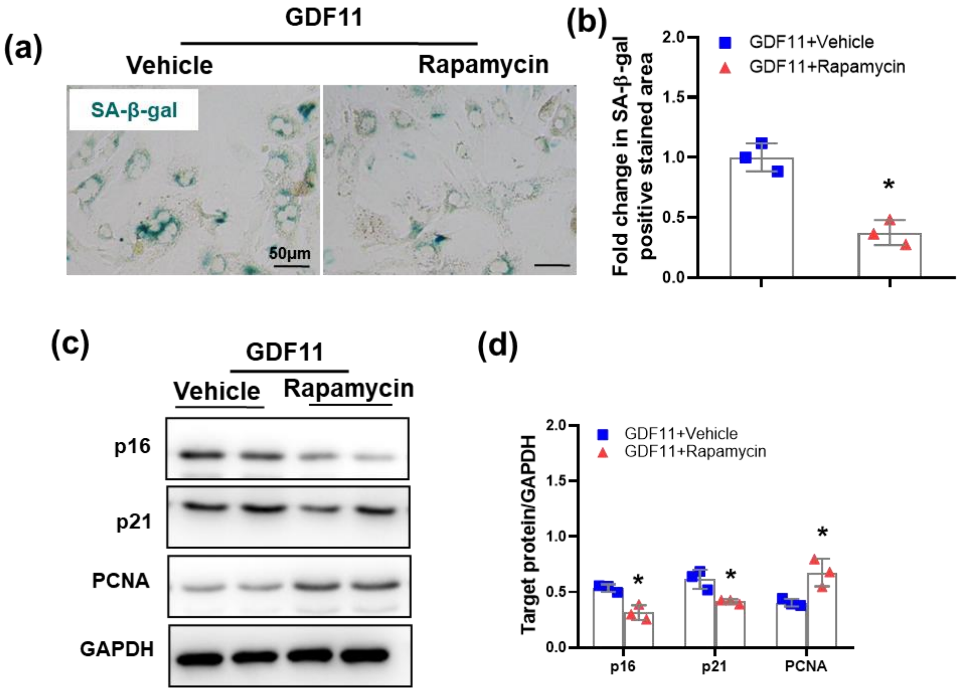

FIGURE S5. GDF11 induces hepatocytes senescence via impairment of autophagic activity. Primary hepatocytes were isolated from aged mice (20 months) and treated with GDF11 (100 ng/ml) in the absence or presence of rapamycin (0.1μM) for 48 hours. (a) Representative micrographs of SA-β-gal staining in hepatocytes (original magnification, 400 x). (b) Percentage of SA-β-gal-positive stained cell area normalized to vehicle controls. (c) Western blot analysis of p16, p21 and PCNA protein expression. (d) Densitometric analysis of p16, p21 and PCNA. The experiment was performed in triplicate with similar results. The data are shown as mean ± SD, *$p<0.05$ compared to the vehicle group.

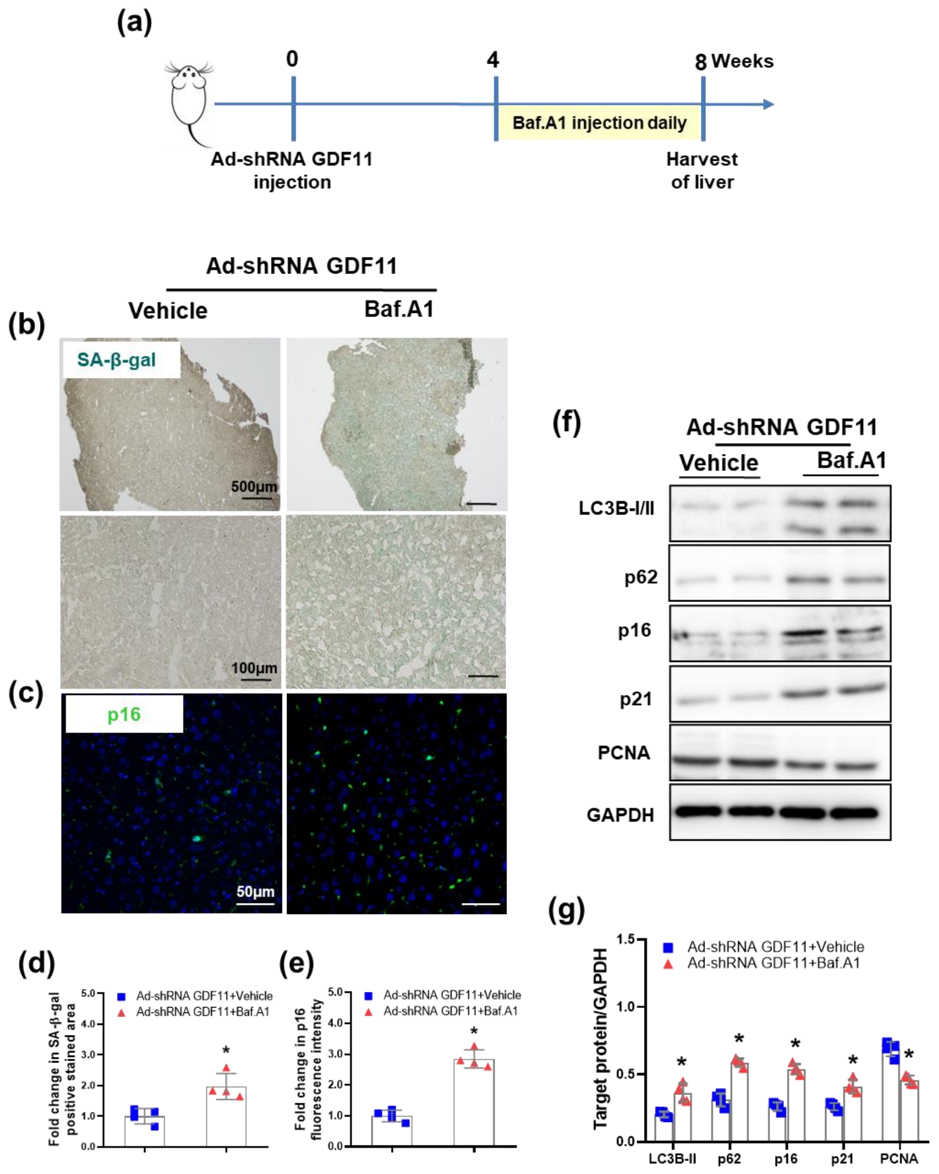

FIGURE S6. Knockdown of GDF11 slows liver senescence via improvement autophagic activity. (a) Experimental timeline: Aged male C57BL/6J mice (20 months) were injected intravenously with a single dose of Ad-shRNA GDF11 (1×10^9 PFU/mouse) on week 0. Bafilomycin A1 (Baf.A1, 1mg/kg, intraperitoneally) was given daily to the Ad-shRNA GDF11-treated aged mice from week 4. On week 8 the mice were sacrificed and livers were harvested as indicated in the layout. (b) Representative micrographs of SA-β-gal staining in hepatic tissues (original magnification, 40 x or 400 x). (c) Representative images of immunofluorescent staining for p16 in hepatic tissues (original magnification, 400 x). (d) Percentage of

SA-β-gal-positive stained cell area normalized to vehicle controls. (e) p16 fluorescence intensity normalized to vehicle controls. (f) Western blot analysis of LC3B, p62, p16, p21 and PCNA protein expression in livers. (g) Densitometric analysis of LC3B-II, p62, p16, p21 and PCNA. The data are shown as mean ± SD, n = 4 per group, *p<0.05 compared to the vehicle group.

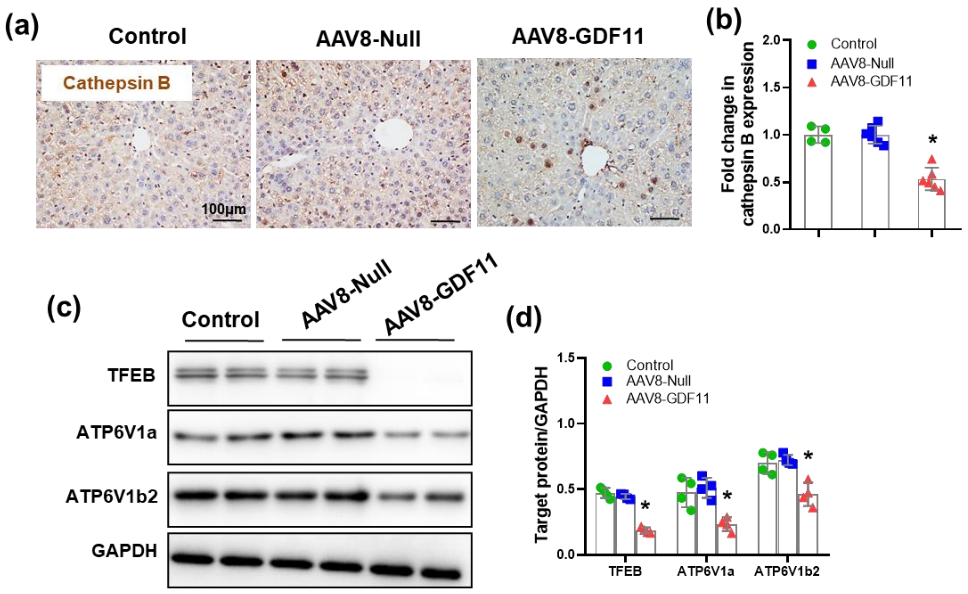

FIGURE S7. Overexpression of GDF11 impairs lysosomal biogenesis. Aged male C57BL/6J mice (20 months) were injected intravenously with a single dose of AAV8-GDF11 (5 x 10^{11} VG/mouse) for 8 weeks. (a) Immunohistochemical staining of liver cathepsin B, and representative images are shown (original magnification, 400 x). (b) Quantification of cathepsin B staining intensity normalized to controls. (c) Total liver lysates were subjected to western blot analysis. (d) Densitometric analysis of TFEB, ATP6V1a and ATP6V1b2. The data are shown as mean ± SD. n=4-6 per group. *p<0.05 compared to the AAV8-Null group.

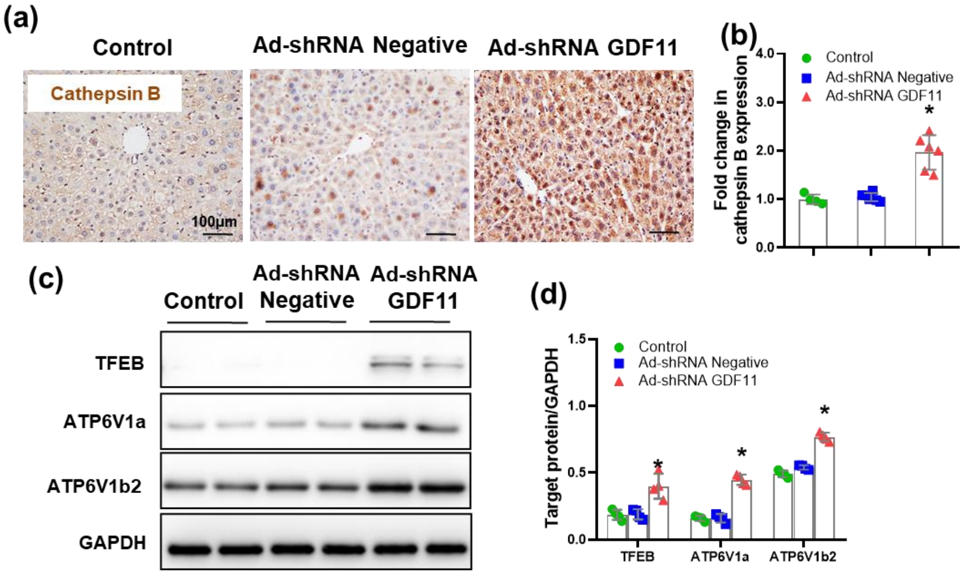

FIGURE S8. Knockdown of GDF11 improves lysosomal biogenesis. Aged male C57BL/6J mice (20 months) were injected intravenously with a single dose of Ad-shRNA GDF11 (1×10^9 PFU/mouse mouse) for 8 weeks. (a) Representative micrographs showing cathepsin B in hepatic tissues (original magnification, 400 x). (b) Quantification of cathepsin B staining intensity normalized to controls. (c) Total liver lysates were subjected to western blot analysis. (d) Densitometric analysis of TFEB, ATP6V1a and ATP6V1b2. The data are shown as mean ± SD. n=4-6 per group. *$p<0.05$ compared to the Ad-shRNA Negative group.

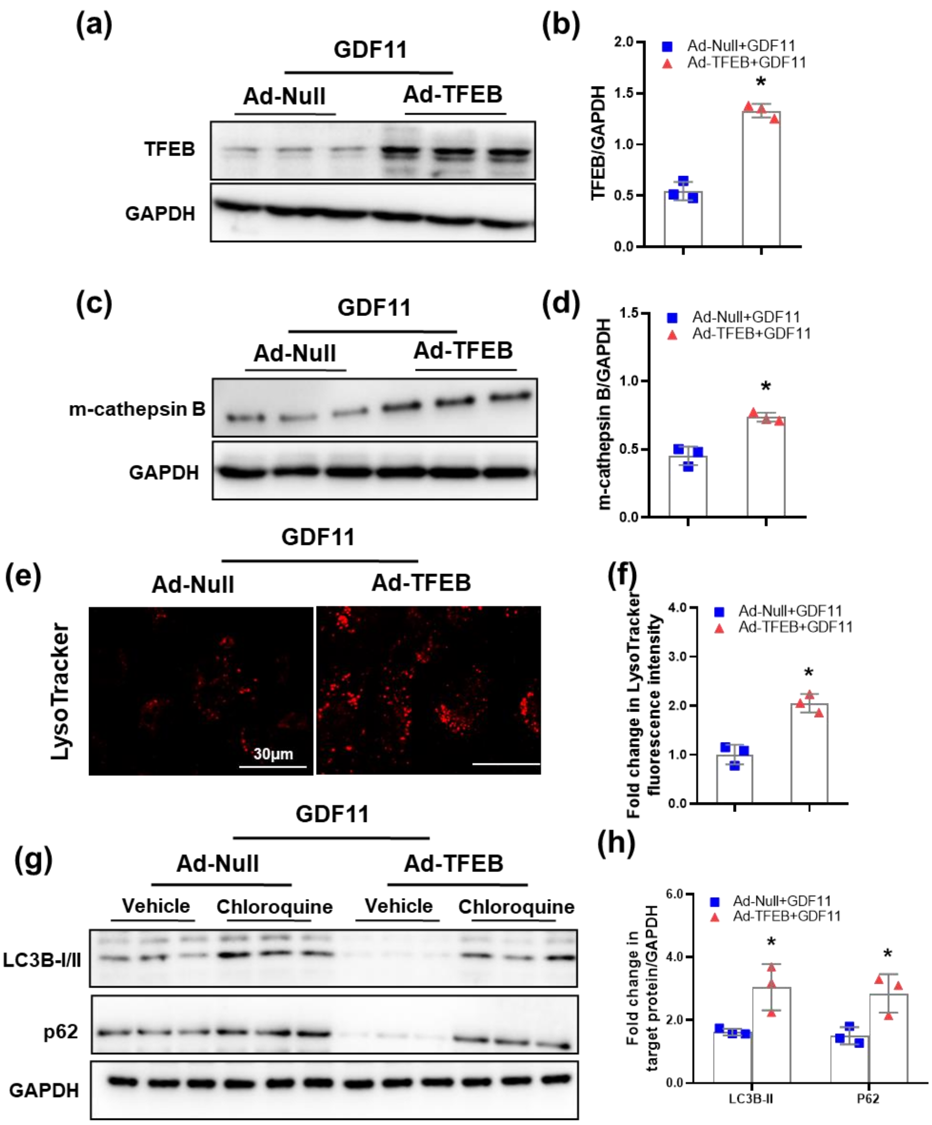

FIGURE S9. Overexpression of TFEB rescues GDF11-impaired lysosomal biogenesis and autophagy. (a) AML12 cells were transfected with Ad-Null or Ad-TFEB and then cultured for 48 hours in presence of GDF11 (100 ng/ml). Western blot analysis of TFEB protein expression. (b) Densitometry analysis of TFEB. (c) Western blot analysis of m-cathepsin B protein expression. (d) Densitometry analysis of m-cathepsin B. (e) AML12 cells were stained with LysoTracker Red DND-99, and then examined by confocal microscopy. Representative images of LysoTracker staining in AML12 cells overexpressed with TFEB (original magnification, 400 x). (f) LysoTracker signal intensity normalized to vehicle controls. (g) Western blot analysis of LC3B and p62 protein expression with or without chloroquine (10μM). (h)

Densitometric analysis of LC3B-II/GAPDH and p62/GAPDH ratio normalized to vehicle controls. The experiment was performed in triplicate with similar results. The data are shown as mean ± SD, *p<0.05 compared to the vehicle group.

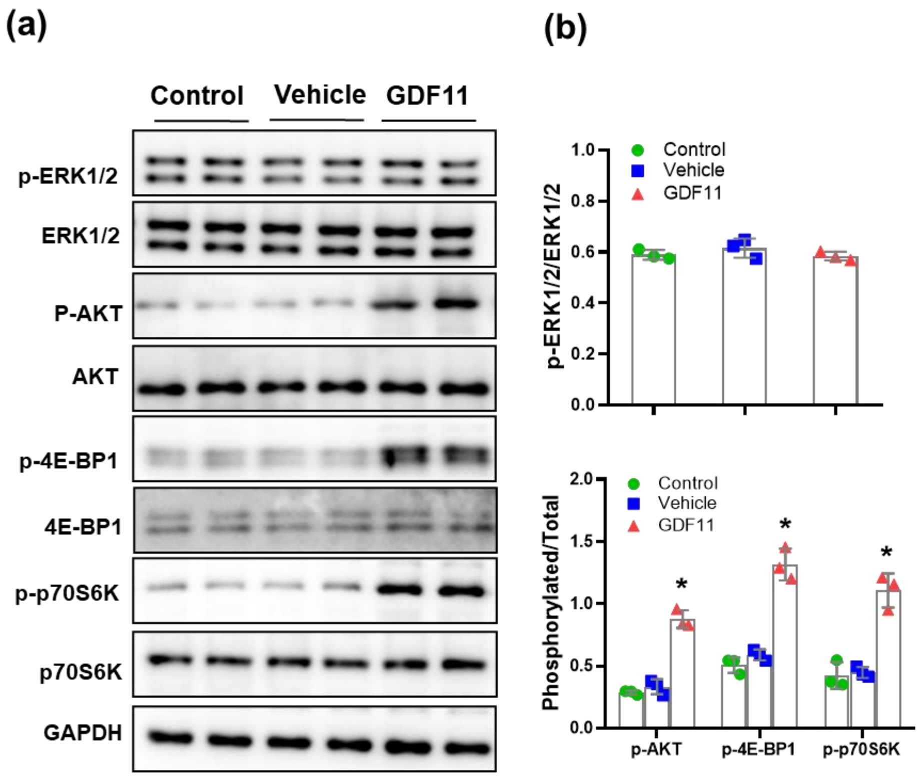

FIGURE S10. GDF11 activates mTORC1 signaling. Primary hepatocytes were isolated from aged mice (20 months) and cultured for 48 hours in presence of GDF11 (100 ng/ml). (a) The protein expression of phosphorylation and total ERK1/2, AKT, 4E-BP1 and p70S6K was examined by immunoblot analysis. (b) Densitometry analysis of p-ERK1/2, p-AKT, p-4E-BP1 and p-p70S6K. The experiment was performed in triplicate with similar results. The data are shown as mean ± SD, *p<0.05 compared to the vehicle group.

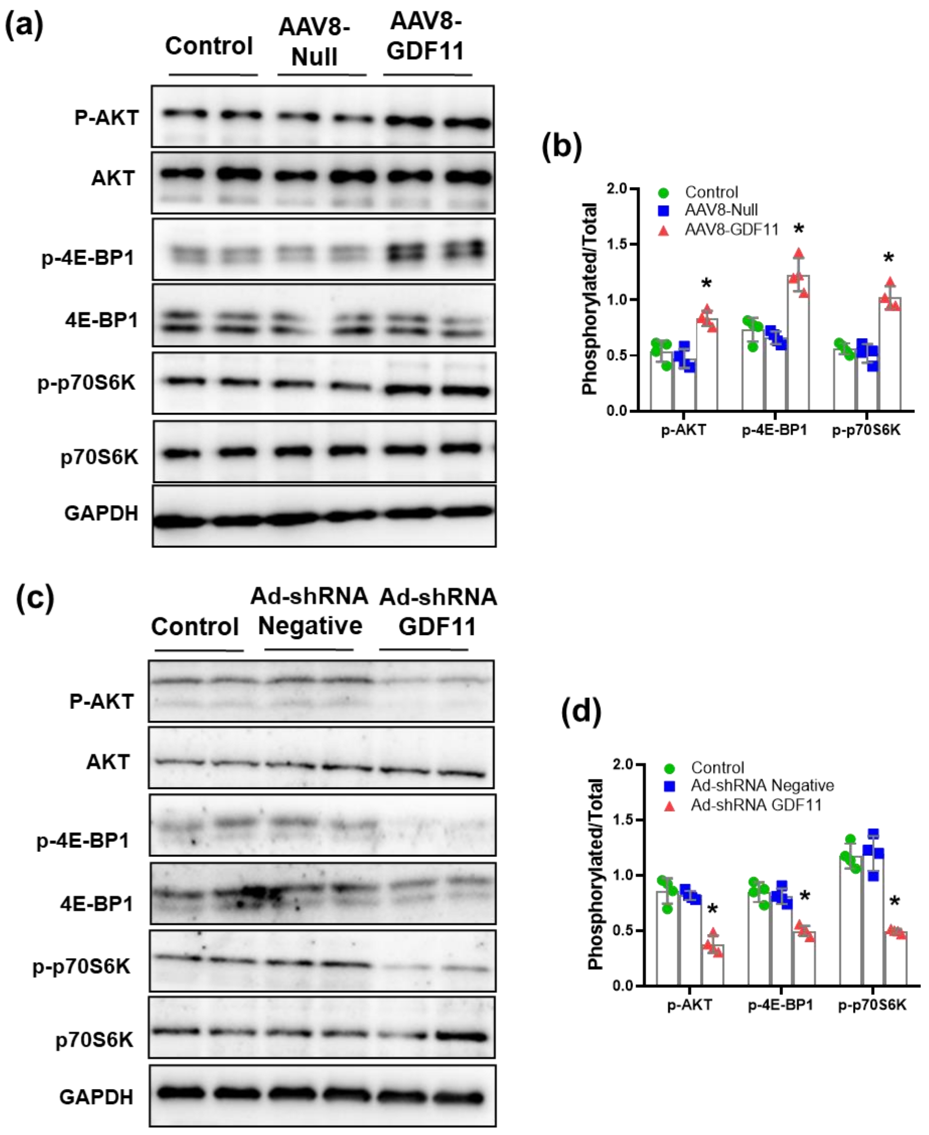

FIGURE S11. Analysis of mTORC1 signaling in the liver from AAV8-GDF11- or Ad-shRNA GDF11-injected mice. Aged male C57BL/6J mice (20 months) were injected intravenously with a single dose of AAV8-GDF11 (5 x 10^{11} VG/mouse) or Ad-shRNA GDF11 (1×10^{9} PFU/mouse mouse) and sacrificed after 8 weeks. (a, c) Total liver lysates were subjected to western blot analysis. (b, d) Densitometric analysis of p-AKT, p-4E-BP1 and p-p70S6K. The data are shown as mean ± SD. n=4 per group. *p<0.05 compared to the AAV8-GDF11 or Ad-shRNA Negative group.

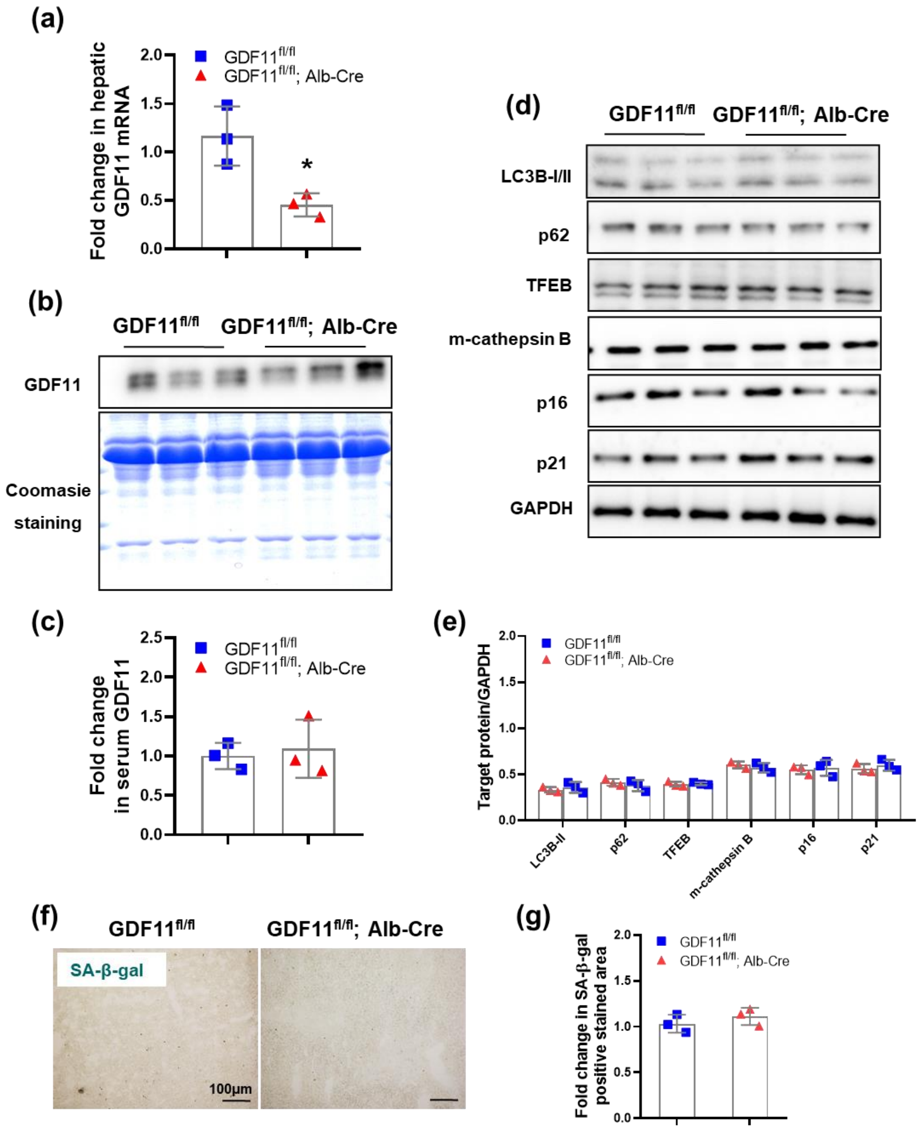

FIGURE S12. Targeted hepatocyte deletion of GDF11 has no effect on lysosomal biogenesis, autophagy and liver senescence. GDF11$^{fl/fl}$ mice and GDF11$^{fl/fl}$; Alb^{Cre} mice (12 months) were sacrificed. (a) qRT-PCR analysis of hepatic GDF11 mRNA expression. (b) Western blot analysis of serum GDF11 protein expression; Coomassie staining (bottom) demonstrates equivalent loading of each lane. (c) Densitometric analysis of serum GDF11 normalized to GDF11$^{fl/fl}$ mice. (d)Total liver lysates were subjected to western blot analysis. (e) Densitometric analysis of LC3B-II, p62, TFEB, m-cathepsin B, p16 and p21. (f) Representative micrographs showing SA-β-gal activity in hepatic tissues (original magnification, 400 x). (g) Percentage of SA-β-gal-positive stained cell area normalized to GDF11$^{fl/fl}$ mice. The data are shown as mean ± SD. n=3 per group. *$p<0.05$ compared to the GDF11$^{fl/fl}$ group.
